# Broad-spectrum antimicrobial activities of a food fermentate of *Aspergillus oryzae*

**DOI:** 10.1128/spectrum.01854-24

**Published:** 2024-10-22

**Authors:** Dasol Choi, Ahmad F. Alshannaq, Yohan Bok, Jae-Hyuk Yu

**Affiliations:** 1 Department of Food Science, University of Wisconsin-Madison, Madison, Wisconsin, USA; 2 Food Research Institute, University of Wisconsin-Madison, Madison, Wisconsin, USA; 3 Department of Bacteriology, University of Wisconsin-Madison, Madison, Wisconsin, USA; 4 School of Medicine and Public Health, University of Wisconsin-Madison, Madison, Wisconsin, USA; Universidade de Sao Paulo, Ribeirao Preto, Sao Paulo, Brazil

**Keywords:** foodborne pathogens, ESKAPE pathogens, antifungal activity, food-fermenting fungus, broad-spectrum antimicrobial, global food safety

## Abstract

Amid persistent concerns over microbial foodborne illnesses and escalating antibiotic resistance, we introduce “NP,” a novel and effective broad-spectrum **n**atural antimicrobial **p**roduct derived from the filtered culture broth of *Aspergillus oryzae* grown in a food-grade liquid medium. NP demonstrates potent bactericidal activity against a range of food-borne and ESKPAE pathogens, including *Staphylococcus aureu*s (including eight distinct drug-resistant methicillin-resistant *Staphylococcus aureus* strains), *Listeria monocytogenes*, *Salmonella typhimurium*, *Klebsiella pneumonia*, *Pseudomonas aeruginosa,* and *Escherichia coli* (including O157:H7) with minimal inhibitory strength ranging from 25% to 100%. In addition, NP exhibits robust antifungal activity against several human pathogenic fungi including *Aspergillus fumigatus*, *Candida albicans,* and the prevalent food spoilage mold *Penicillium* species, arresting spore germination and vegetative cell growth. Mechanistically, NP disrupts the structural integrity of bacterial and fungal cell membranes, increasing membrane permeability and leading to cell death. Furthermore, genome-wide expression analyses of *A. fumigatus* vegetative cells exposed to NP reveal the downregulation of genes associated with the liveness of the fungal cells including ergosterol biosynthesis, cell wall maintenance, and development, with network analysis highlighting NP’s impact on various metabolic pathways. Notably, NP is presumed safe and thermally stable, holding promise for addressing foodborne illnesses and drug-resistant infections through the development and widespread application of a new generation of antimicrobials.

**IMPORTANCE:**

The development of NP, a potent broad-spectrum antimicrobial, is a significant breakthrough in the ongoing challenge against microbial foodborne illnesses and the growing threat of antibiotic resistance. This food-grade culture broth of *Aspergillus oryzae* demonstrates exceptional broad-spectrum efficacy against a variety of harmful bacteria and fungi, including drug-resistant strains such as methicillin-resistant *Staphylococcus aureus* and prevalent food spoilage molds. NP exhibits strong bactericidal activity against various foodborne and ESKAPE pathogens, and strong antifungal activity against Penicillium species, *Aspergillus fumigatus*, and *Candida albicans*. The potent bactericidal and antifungal properties of NP are a result of its ability to disrupt microbial cell membranes leading to increased permeability. Furthermore, the genome-wide impact of NP on fungal gene expression and metabolic pathways underscores its comprehensive antimicrobial action, leading to transcriptomic and metabolic changes associated with cell death in *A. fumigatus*.

## INTRODUCTION

In the past decade, there has been a surge in global concern about the escalating antimicrobial resistance and foodborne illness. The Centers for Disease Control (CDC) report on Antibiotic Resistance Threats in the United States (1) underscores this concern, revealing an annual incidence of over 2.8 million cases of antibiotic-resistant infections in the United States alone, leading to more than 35,000 deaths (1). In addition, the economic impact is substantial, with an estimated annual cost of $17 billion attributed to foodborne illnesses in the United States (2). Recent data from the European Union (EU) present an alarming trend, with a significant 44% increase in reported foodborne outbreaks in 2022 compared to the previous year (3). Furthermore, the rise in infections and mortality associated with multidrug-resistant ESKAPE pathogens (including *Enterococcus faecium*, *Staphylococcus aureus*, *Klebsiella pneumoniae*, *Acinetobacter baumannii*, *Pseudomonas aeruginosa*, and *Enterobacter* species) in Europe from 2016 to 2020 underscores the urgent need for effective antimicrobial strategies (4, 5). Fungal infections, particularly those stemming from antimicrobial-resistant species such as *Aspergillus* and specific *Candida* strains, pose significant challenges to global health, impacting over one billion individuals worldwide and contributing to conditions such as chronic pulmonary aspergillosis and invasive candidiasis (6).

Given the gravity of antimicrobial resistance, exploring alternative antimicrobial agents is imperative, and natural antimicrobials derived from microorganisms offer promising solutions across various sectors, including agriculture, industry, and medicine (7). Filamentous fungi (molds) have a long-standing history of serving as a source of organic acids, proteins, enzymes, and small-molecule drugs, including antibiotics, statins, and steroids (8). Many *Aspergillus* species are known for their capacity to produce antibacterial metabolites, prompting exploration as potential alternatives to conventional antibiotics (9, 10). Of significance is the genus *Aspergillus*, which encompasses three koji molds—*Aspergillus oryzae*, *Aspergillus sojae*, and *Aspergillus luchuensis*—certified by the Brewing Society of Japan (11, 12).

**Fig 1:**
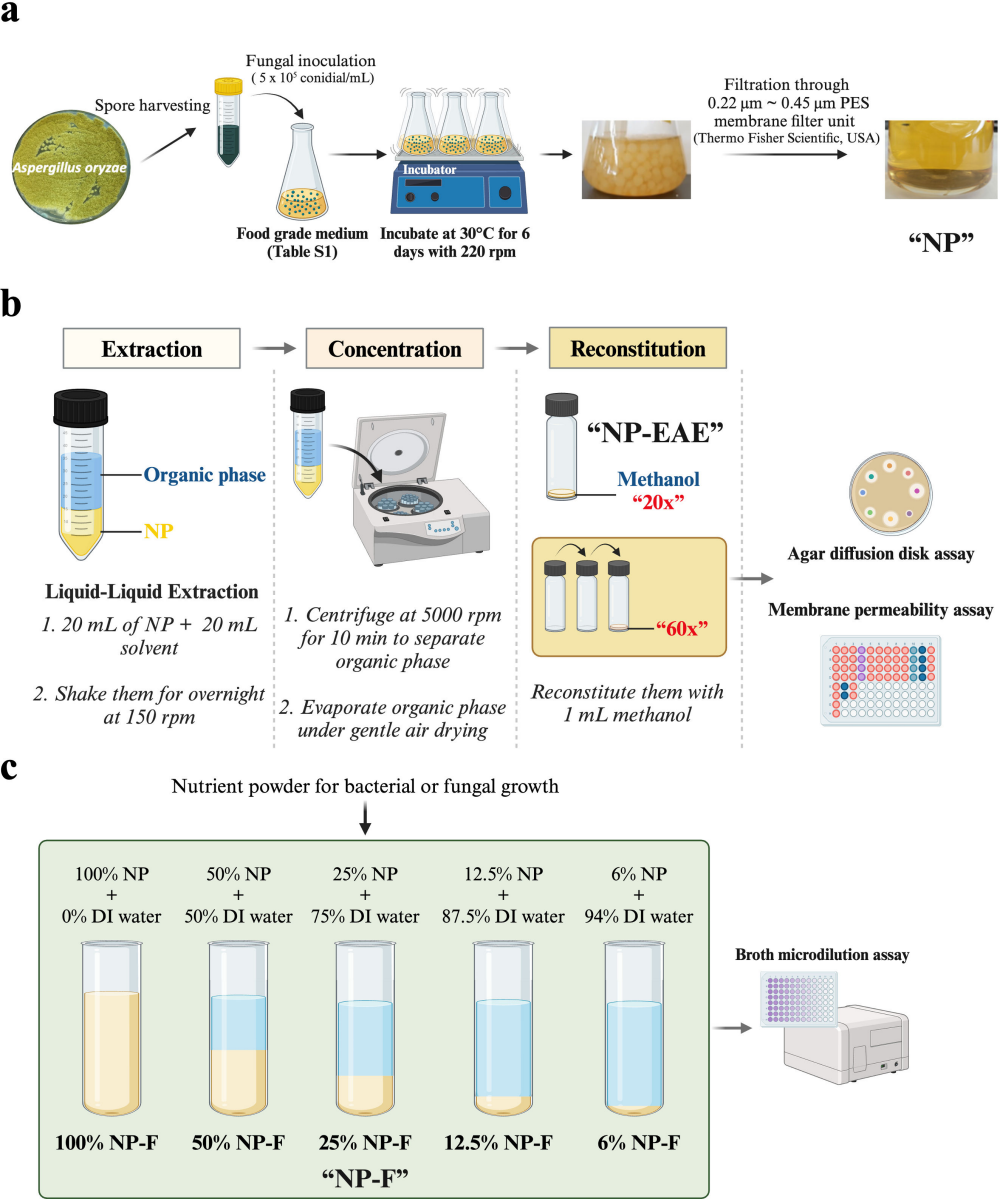
Schematic presentation of production of NP, NP-EAE, and NP-F. The process of producing NP (a), NP-EAE (b), and NP-F (c) is illustrated in this schematic, outlining the steps involved in each production method. Figures were created with Biorender.com.

In response to this urgent global concern, we have developed a novel food-grade broad-spectrum antimicrobial product, designated “NP” (Natural Product), utilizing the Generally Recognized as Safe (GRAS) fungus *A. oryzae*. NP originates from this safe fungus grown in a liquid medium comprising edible components followed by filtration of the resulting culture broth (see Fig. 1a). Our findings demonstrate NP’s efficacy in inhibiting the growth of foodborne pathogens, ESKAPE pathogens, opportunistic human pathogenic fungi, and food spoilage fungi. Analyses of the bacterial and fungal membrane permeability, fungal transcriptomic responses, and examination of protein-protein interaction networks in actively growing *Aspergillus fumigatus* cells have led to a potential mechanism underlying the broad-spectrum antimicrobial properties. Our findings underscore NP’s potential as a viable substitute for antibiotics in treating infections, thereby substantially contributing to global health security.

## RESULTS

### Antibacterial activity of NP-EAE and NP-F

#### NP effectively inhibits the growth of food-born and ESKAPE bacterial pathogens

To produce NP, we tested 11 different media under various growth conditions, as detailed in Table S7. Our results showed that culturing *A. oryzae* NRRL 3483 in TSB and BHI media provided the best antimicrobial activity against both *S. aureus* S6 (Fig. S1a) and *E. coli* K12 FRIK 2637 (Fig. S1b). However, since BHI (Brain Heart Infusion) contains beef heart and calf brain extracts and is not considered a food-grade medium, we focused on TSB. Culturing *A. oryzae* NRRL 3483 in TSB medium, which contains glucose, a pancreatic digest of casein, and papain digest of soybean, for 6 days at 30°C and 220 rpm, yielded a potent NP (Fig. S1).

Various organic solvents were evaluated for their ability to extract antimicrobial compounds from NP. Ethyl acetate extract (NP-EAE) demonstrated significant inhibition against *S. aureus* S6, whereas toluene and BEA extracts showed no inhibition. Consequently, ethyl acetate was selected as the optimal solvent (Fig. S2a), highlighting the importance of polar compounds in NP’s antimicrobial activity. A dose-response assessment of NP-EAE against *S. aureus* S6 revealed that a 100 µL loading volume was optimal, producing an inhibition zone of approximately 20 mm (Fig. S2b).

**Fig 2:**
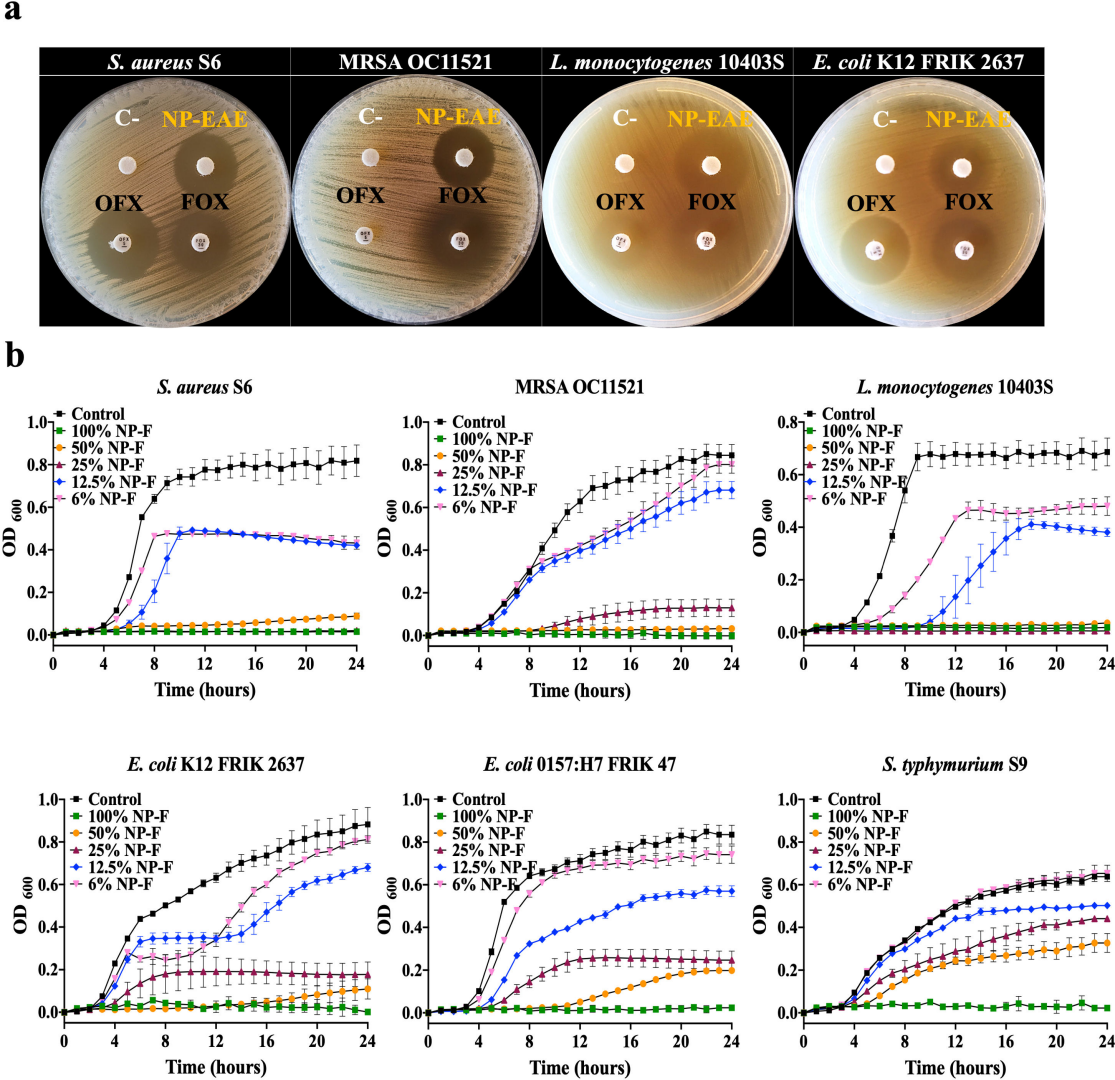
Antibacterial activities of NP-EAE and NP-F. (a) Zone of inhibition caused by NP-EAE against *S. aureus* S6, MRSA OC11521, *L. monocytogenes* 10403S, and *E. coli* K12 FRIK 2637. Disks were loaded with 100 µL of the NP-EAE (top right disk). A disk loaded with 5 µg ofloxacin (OFX 5; bottom left disk) and 30 µg of cefoxitin (FOX 30; bottom right disk) were used as the positive controls. Methanol was used as the negative control (C-; top left). (b) Growth curves of *S. aureus* S6, MRSA OC11521, *L. monocytogenes* 10403S, *E. coli* K12 FRIK 2637, *E. coli* O157:H7 FRIK 47, and *S. typhimurium* S9 in 100%, 50%, 25%, 12.5%, and 6% NP-F (MHB medium) at 37°C for 24 hours. The original MHB medium (0% NP-F) was used as the negative control. The experiment was performed in triplicate (*n* = 3).

The antibacterial effects of NP-EAE were assessed using disk diffusion tests against several foodborne pathogens, including *S. aureus* S6, methicillin-resistant *Staphylococcus aureus* (MRSA) OC11521, *L. monocytogenes* 10403S, and *E. coli* K12 FRIK 2637. NP-EAE exhibited substantial antibacterial activity, comparable to or even surpassing 5 µg of ofloxacin or 30 µg of cefoxitin (Fig. 2a). Further validation in Fig. S3 showed the antibacterial performance of NP-EAE through the growth curves of *S. aureus* S6, *L. monocytogenes* 10403S, *E. coli* K12 FRIK 2637, and *S. typhimurium* S9. By contrast, the autoclaved medium without fungal inoculation did not show significant bioactivity under identical testing conditions. Moreover, the heat stability of NP’s antimicrobial compounds suggests they are resistant to thermal degradation. These findings reinforce that the observed bioactivity is due to the metabolic activity of *A. oryzae*, rather than any byproducts of the autoclaving process.

For a direct assessment of NP’s efficacy without the extraction process, NP formulated with rich nutrient medium (NP-F) was used. Minimum inhibitory concentrations (MICs) were determined as a percentage (%) of NP-F, with 50% NP-F for *S. aureus* S6 and MRSA OC11521, 25% for *L. monocytogenes* 10403S, and 100% for *E. coli* K12 FRIK 2637, 0157:H7 FRIK 47, and *S. typhimurium* S9 (Fig. 2b). NP-F was also tested against antibiotic-resistant bacteria, including MRSA (known for methicillin and β-lactam resistance) and ESKAPE pathogens (notorious for evading antimicrobials) (13, 14). As shown in Fig. S4, NP-F effectively inhibited the growth of eight MRSA strains even at 50% concentration. In addition, 100% NP-F completely halted the growth of two antibiotic-resistant *S. aureus* strains, MW2 and 33593 (Fig. S5a), and impeded the growth of *K. pneumoniae* 81-1269A (Fig. S5b). It also showed inhibition against *P. aeruginosa* strains 2368, 3060, and 65 to some extent (Fig. S5c), as well as *E. coli* 1-894-1 (Fig. S5d). This demonstrates NP’s efficacy against antibiotic-resistant foodborne and ESKAPE pathogens, highlighting significant antibacterial activity across various bacterial strains.

#### NP-F efficiently kills bacterial pathogens

**Fig 3:**
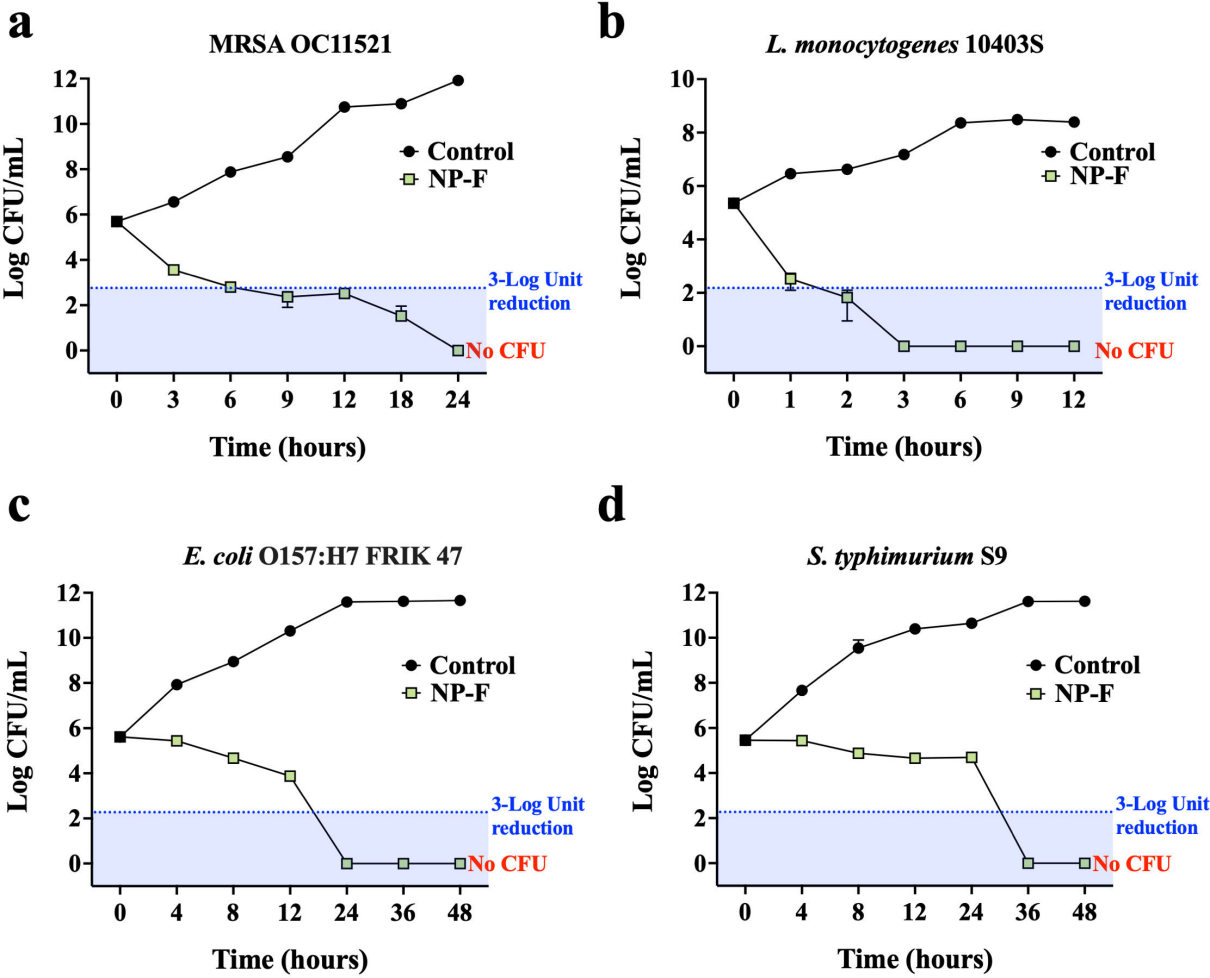
Time-dependent bactericidal activity of NP-F. CFU of log-phase MRSA OC11521 (a), *L. monocytogenes* 10403S (b), *E. coli* O157:H7 FRIK 47 (c), and *S. typhimurium* S9 (d) incubated in 100% NP-F MHB liquid medium at 37°C for 12–48 hours. The original MHB medium was used as the negative control. The experiment was performed in triplicate (*n* = 3).

To determine whether NP-mediated antibacterial activity is bactericidal or bacteriostatic, we carried out time-kill experiments against MRSA OC11521, *L. monocytogenes* 10403S, *E. coli* O157:H7 FRIK 47, and *S. typhimurium* S9. In 100% NP-F (MHB liquid medium), MRSA OC11521 exhibited a 3-log reduction within 6 hours, with no detectable live cells at 24 hours (Fig. 3a). In addition, NP-F eradicated 100% of *L. monocytogenes* cells within 3 hours, achieving a 3-log reduction within 1 hour (Fig. 3b). For Gram-negative bacteria, NP-F eliminated all *E. coli* cells within 24 hours and all *S. typhimurium* cells within 36 hours (Fig. 3c and d). These findings demonstrate the effective bactericidal activity of NP-F against both Gram-positive and Gram-negative bacterial pathogens. Based on the rapid killing of bacterial cells, we hypothesize that NP effectively eliminates bacterial pathogens by disrupting their cell membranes.

### NP disrupts bacterial cell membranes

**Fig 4:**
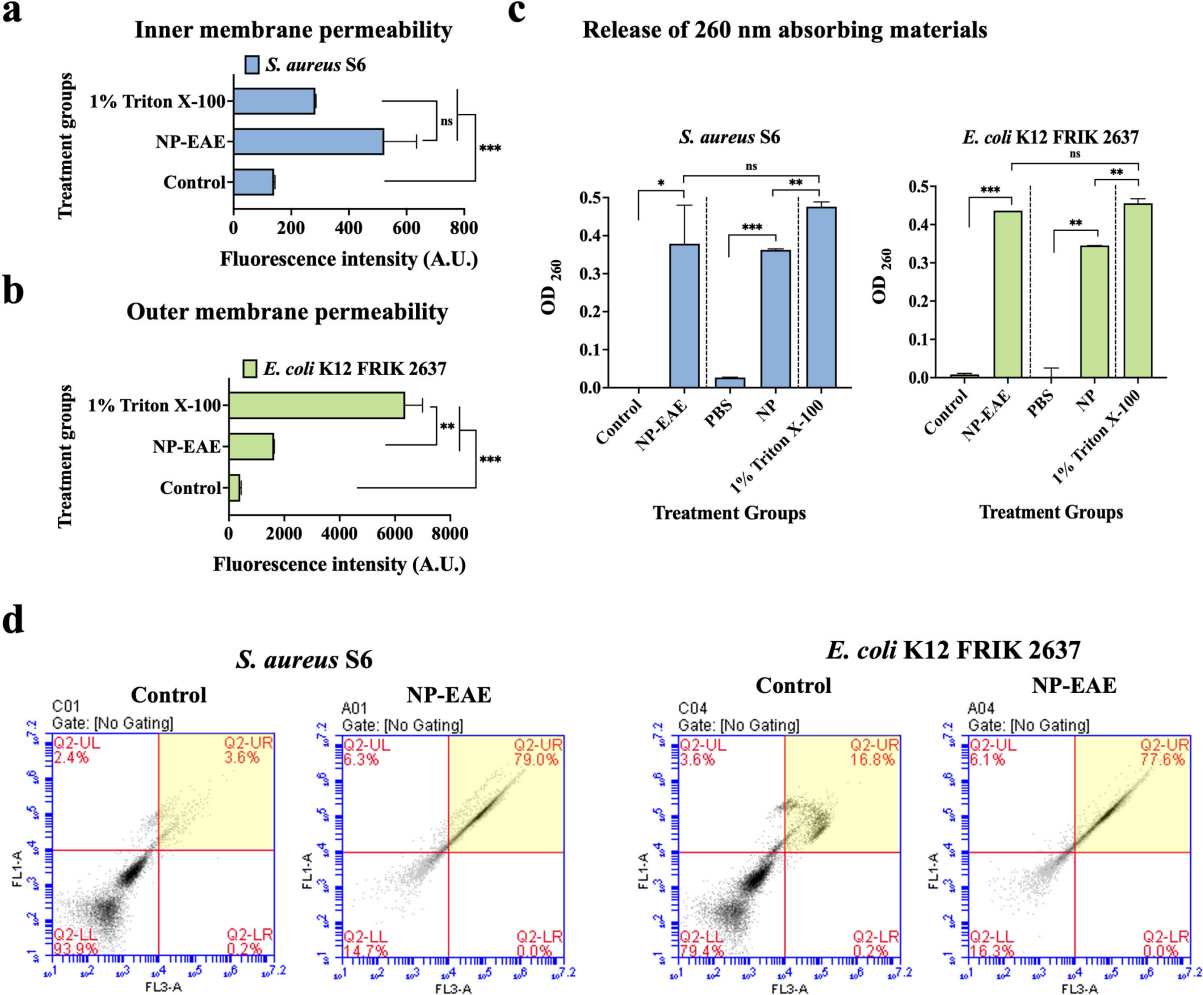
Potential mechanism of bactericidal activity attributed to NP. (a) Inner-membrane permeability of the Gram-positive bacteria *S. aureus* S6 and (b) cell outer membrane permeability of the Gram-negative bacteria *E. coli* K12 FRIK after 6 hours of exposure to NP-EAE. (c) The release of cellular bio-materials absorbing 260 nm wavelength from bacterial cells after 3 hours of exposure to NP-EAE and NP. (d) Flow cytometry analysis of NP-EAE after 6 hours of exposure to NP-EAE, where the X-axis (FL3-A) represents the fluorescence intensity of the PI dye, and the Y-axis (FL1-A) shows the fluorescence intensity of the Syto-9 dye on a log scale. The highlighted yellow regions (top right corner) denote high-intensity PI fluorescence. Positive controls included Triton-X 100 (1%), while negative controls were PBS buffer and methanol. *P < 0.05; **P < 0.01; ***P < 0.001; ns not significant. The experiment was performed in triplicate (*n*= 3).

To test the hypothesized correlation between membrane permeabilization and bacterial viability, we conducted experiments to determine whether NP’s impact on bacterial death is due to the direct disruption of the inner membrane of *S. aureus* S6 and the outer membrane of *E. coli* K12 FRIK 2637. After 6 hours of NP-EAE treatment, there were significant increases in SYTOX Green fluorescence intensity (Fig. 4a), a DNA-binding dye that is normally impermeable to intact membranes (15), and NPN fluorescence intensity (Fig. 4b), a neutral hydrophobic fluorescent probe typically restricted by the outer membrane (16). These results suggest that the bioactive compounds present in NP can rapidly permeabilize bacterial cellular membranes.

Furthermore, the release of intracellular components such as potassium, phosphate, nucleic acids, and other cellular materials was monitored to confirm membrane disruption in *S. aureus* and *E. coli*. Even after just 3 hours of NP-EAE treatment, a significant release of these intracellular components was observed, indicating NP’s rapid membrane-disrupting effects (Fig. 4c). Flow cytometry was used to further evaluate the damaging effects of NP-EAE on the membrane. As shown in Fig. 4d, 79.0% of *S. aureus* and 77.6% of *E. coli* cells exhibited membrane permeabilization, compared to less than 20% in the negative control (methanol). These results indicate that NP induces significant disruption of membrane integrity in bacterial pathogens.

### Antifungal activity of NP

#### NP effectively inhibits the growth of food spoilage and pathogenic fungi

**Fig 5:**
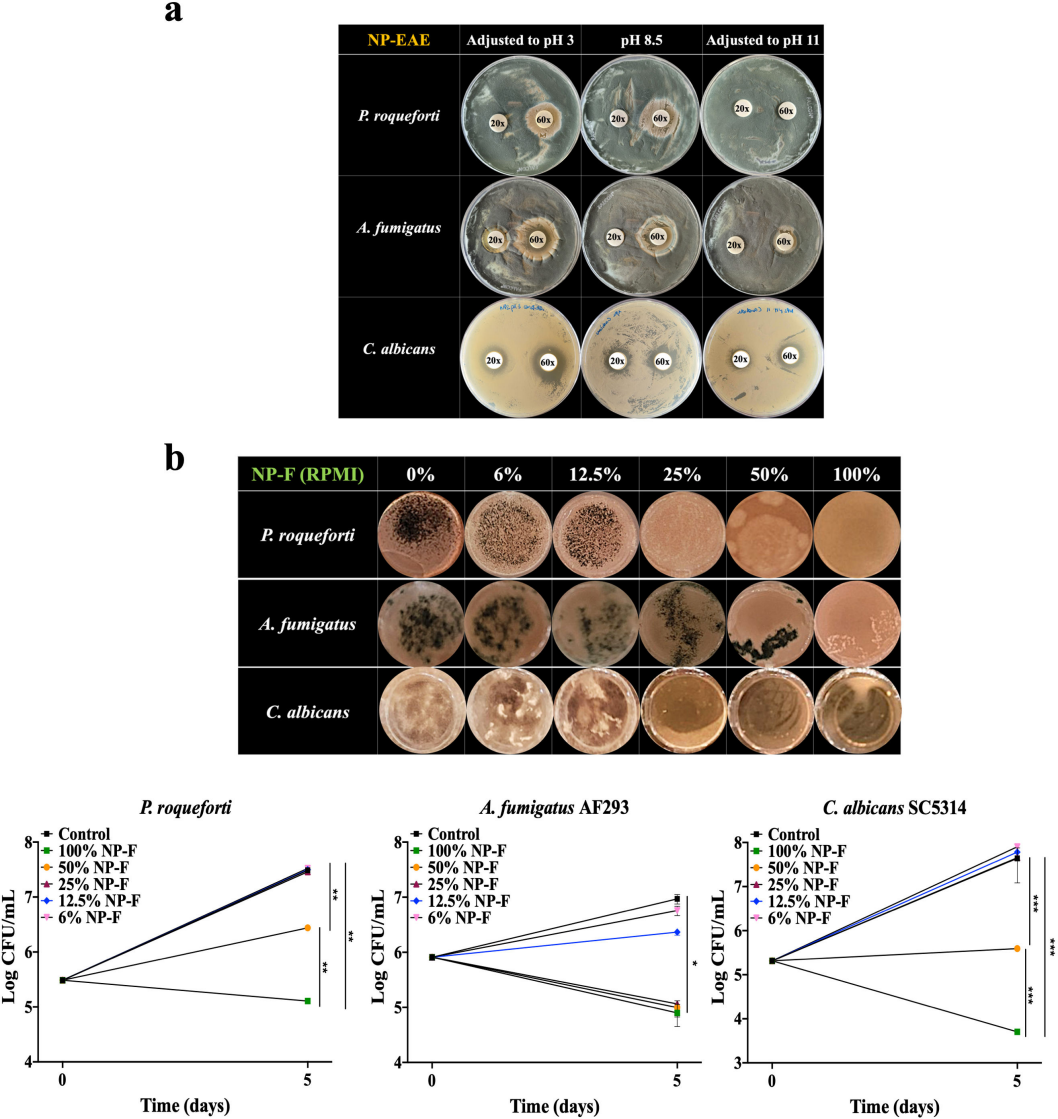
Antifungal activities of NP-EAE and NP-F. (a) Zone of inhibition against *P. roqueforti*, *A. fumigatus* AF293, and *C. albicans* SC5314 produced by 20× and 60× equivalents of NP-EAE. NP-EAE was prepared after NP was adjusted to pH 3, 8.5 (original pH), and 11. A total of 300 µL of each extract was applied to 13 mm sterile paper disks and placed on PDA solid medium for *P. roqueforti* and *A. fumigatus*, and YPD solid agar medium for *C. albicans*. (b) Growth of *P. roqueforti* at 25°C, *A. fumigatus* AF293 at 37°C, and *C. albicans* SC5314 at 30°C in 2 mL of 100%, 50%, 25%, 12.5%, and 6% NP-F (RPMI liquid medium) in a 24-well plate over 5 days. After incubation, aliquots from each well were plated on PDA solid medium for *P. roqueforti* and *A. fumigatus*, and YPD solid agar medium for *C. albicans*, and CFUs were counted. The RPMI medium without NP-F served as the negative control. Error bars represent the standard deviation of three independent experiments. *P < 0.05; **P < 0.01; ***P < 0.001.

We investigated NP’s potential as an antifungal agent by testing it against the food spoilage fungus *P. roqueforti* and two human opportunistic pathogenic fungi *A. fumigatus* AF293 and *C. albicans* SC5314. To explore NP’s adaptability across different applications, we adjusted its pH from the initial 8.5 to pH3 and pH11 using 10 N NaOH and 6 N HCl, respectively. This adjustment aimed to mimic a range of environmental conditions under which NP could function as an antifungal agent, thus expanding its potential practicality. As shown in Fig. 5a, NP-EAE exhibited enhanced antifungal effectiveness at a concentration of 60×, evidenced by distinct inhibition zones surrounding the 13 mm disks on a PDA medium. Specifically, at pH3, NP-EAE showed a 28.0 ± 1.1 mm inhibition zone against *P. roqueforti*, while at the original pH (~8.5), it achieved a 29.1 ± 1.5 mm inhibition zone, with no inhibition observed at pH 11. For *A. fumigatus*, the inhibition zones were 31.5 ± 2.6 mm (pH 3), 26.4 ± 0.9 mm (pH 8.5), and 17.3 ± 2.6 mm (pH 11). Regarding *C. albicans*, the inhibition zones were 27.0 ± 1.7 mm (pH 3), 21.9 ± 0.8 mm (original pH), and 19.6 ± 0.8 mm (pH 11). These findings underscore NP’s potent antifungal activity across a broad pH spectrum.

To evaluate the persistence of NP’s effectiveness at various dilutions, NP was mixed with distilled water while preserving the same nutrient content required for fungal growth in RPMI liquid medium and subsequently evaluated for its antifungal properties. As shown in Fig. 5b, *P*. *roqueforti*, *A. fumigatus*, and *C. albicans* were exposed to NP-F at concentrations ranging from 25% to 100% in 2 mL RPMI liquid medium. After incubation, spores from the RPMI medium were collected, and CFUs were counted following plating on a solid agar medium. NP-F significantly inhibited spore germination and growth of *P. roqueforti* compared to the control (RPMI medium with 0% NP-F), with even 25% NP-F showing inhibitory effects. Specifically, 100% NP-F resulted in a substantial 2.37 log reduction in *P. roqueforti* growth after 5 days. Similarly, *A. fumigatus* growth and germination were significantly hindered by 100% NP-F, exhibiting a significant 1.98 log difference from the control’s growth after 5 days. In addition, 50% and 100% NP-F blocked the growth *C. albicans*, leading to a remarkable 4.19 log reduction compared to the growth of *C. albicans* in the control group after 5 days.

Furthermore, spore suspensions from various *Penicillium* species, including *P. roqueforti*, *P. chrysogenum*, and *P. expansum*, as well as five strains of *A. fumigatus*, were applied to PDA solid medium having different NP-F concentrations (10%, 25%, 50%, and 100%). Remarkably, the application of 100% NP-F (PDA) resulted in complete inhibition of growth in *Penicillium* species, and even a modest concentration of 10% NP-F (PDA) significantly impeded the growth of all five strains of *A. fumigatus* (Fig. S6). These results imply that dilutions of NP could offer a cost-effective and practical antifungal solution.

### Time-dependent antifungal activity of NP-F and safety of NP

**Fig 6:**
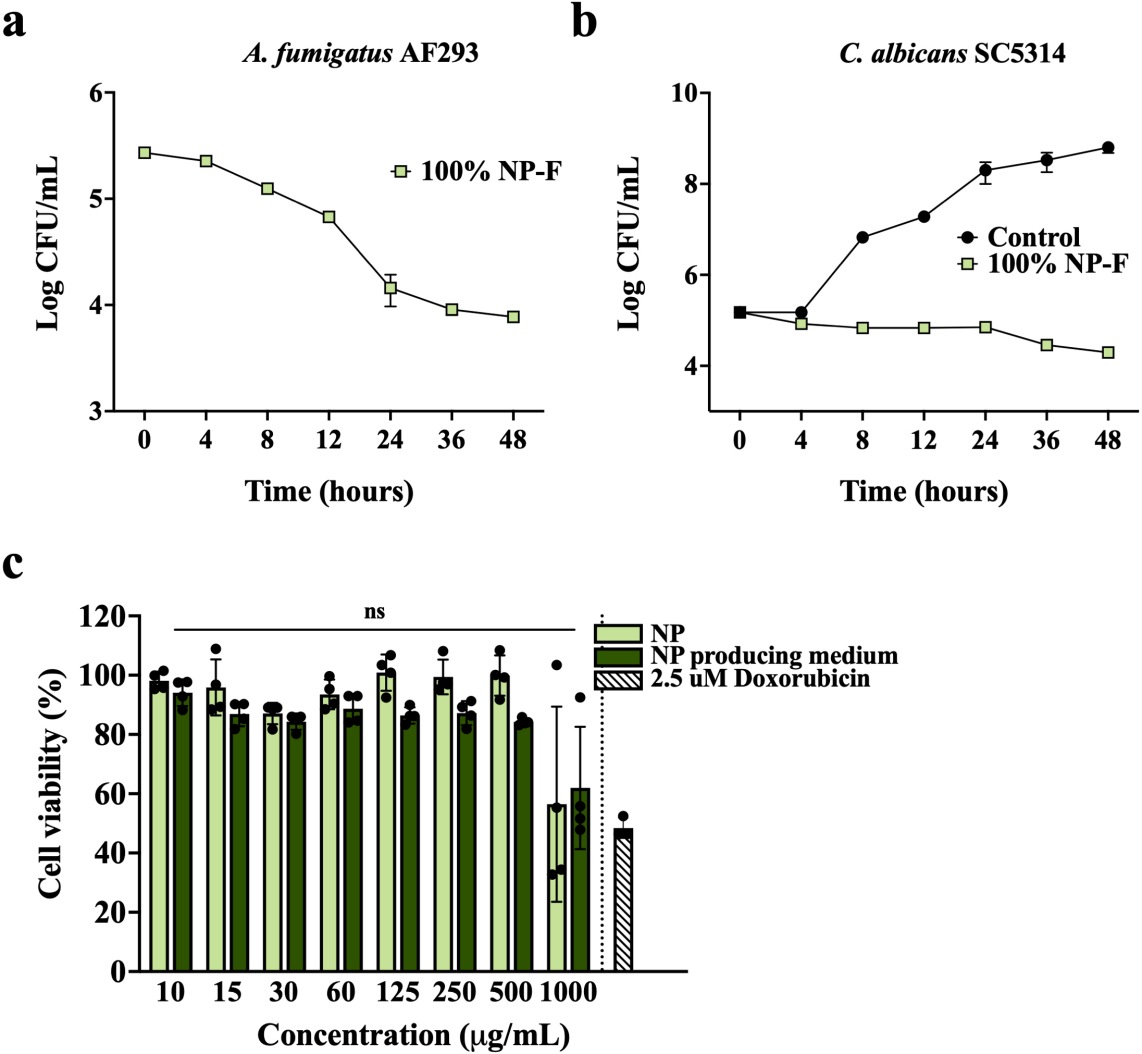
Time-dependent antifungal activity of NP-F and NP’s non-toxic nature. CFU of (a) *A. fumigatus* AF293 and (b) *C. albicans* SC5314 cultured in 100% NP-F (RPMI liquid medium) at 37°C and 30°C, respectively. The original RPMI liquid medium was used as the negative control. (c) Viability of the MCF-7 cells with varying amounts of freeze-dried control and NP. ns not significant. The experiment was performed in triplicate (*n* = 3).

To determine whether NP functions as a fungicidal or fungistatic agent, 5 × 10^5^ spores of *A. fumigatus* and 5 × 10^5^ cells of *C. albicans* were exposed to 100% NP-F RPMI liquid medium for 48 hours. The results revealed a 1.54 log reduction in the viability of *A. fumigatus* spores, while the control group had already undergone germination, leading to mycelia growth that made it challenging to count CFU (Fig. 6a). In addition, there was a 0.88 log reduction in cell viability for *C. albicans* cells, resulting in a substantial 4.50 log difference compared to the control group (Fig. 6b). These findings strongly indicate that NP-F effectively eradicates fungal spores and cells through its fungicidal effect.

Importantly, a toxicity evaluation of NP was conducted using the MCF-7 breast cancer cells, which are highly sensitive to xenobiotics due to their rapid cell division rate. As shown in Fig. 6c, when exposed to up to 500 µg/mL of lyophilized NP, the cells maintained high viability and even at 1000 µg/mL of lyophilized NP, cell viability remained at approximately 60%, similar to that observed in the non-fermented NP-producing medium containing 2.5 g/L of glucose, consistent with prior report on MCF-7 cells’ sensitivity to glucose (17). These results suggest that NP is a fermented food product capable of effectively and safely inhibiting various bacterial and fungal pathogens.

### NP effectively disrupts the cell membrane of *A. fumigatus*

**Fig 7:**
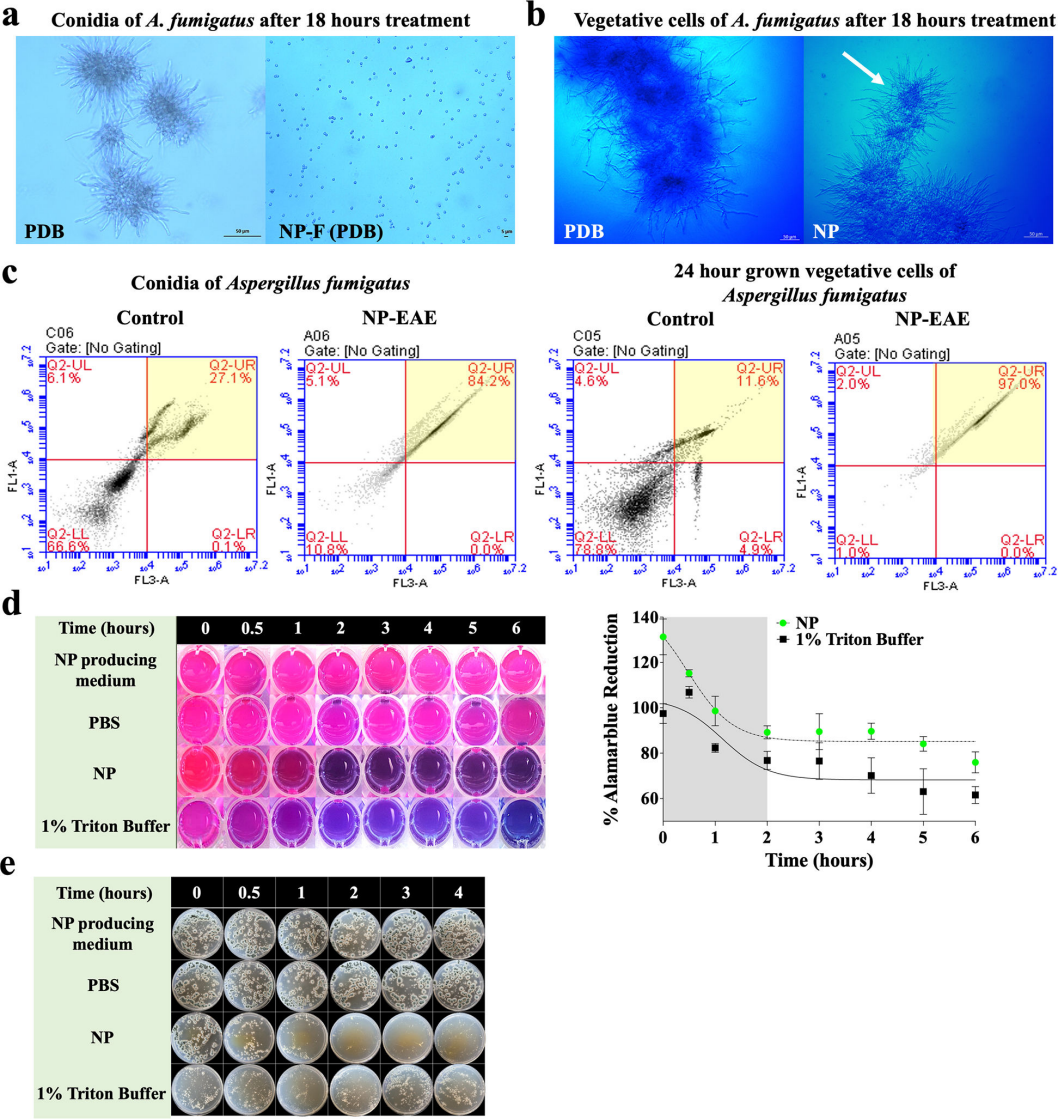
Potential mechanism of fungicidal activity resulting from NP. Antifungal activity of the NP-F and NP against (a) spores and (b) actively growing vegetative cells (mycelia) of *A. fumigatus* AF293. Microscopic images of the fungal cells of *A. fumigatus* after 18 hours of exposure. (c). Cell membrane permeabilization levels of conidia (left) and 24-hour grown vegetative cells of *A. fumigatus* (right) treated with control (methanol, negative control) and NP-EAE for 6 hours were determined by flow cytometry with the membrane-permeable dyes Syto-9 and PI. The X-axis (FL3-A) shows the fluorescence intensity of the PI dye, and the Y-axis (FL1-A) shows the fluorescence intensity of the Syto-9 dye in a log scale. Regions highlighted in yellow (top right corner) show high-intensity PI fluorescence. (d) Cell viability of *A. fumigatus* by color changes (left) and the differences in reduction (%, right) using four different treatments were assessed at 0, 0.5, 1, 2, 3, 4, 5, and 6 hours after inoculation. (e) Plate photographs of mycelia present in 1 mL of individual treatment groups/conditions inoculated on PDA solid medium and incubated at 37°C for 24 hours. PDB and Triton-X 100 (1%) were used as positive controls and methanol, NP-producing medium, PBS, and buffer were used as negative controls. The experiment was performed in triplicate (*n* = 3).

To determine whether NP interferes with the membrane of *A. fumigatus*, indicative of a fungicidal effect, an antifungal activity test was conducted using spores (conidia) and vegetative cells (mycelia) of *A. fumigatus* 293. NP-F completely blocked spore germination, even in the presence of PDB (Fig. 7a). In addition, NP inhibited hyphal growth and led to the formation of aggregated and compacted hyphal pellets, distinct from the control group (Fig. 7b).

Analysis of fungal cell membrane permeability showed that over 84% of conidia and 97% of vegetative cells of *A. fumigatus* 293 exhibited high-intensity PI fluorescence after a 6-hour treatment with NP-EAE (Fig. 7c), indicating greater susceptibility of vegetative cells to the NP-EAE than conidia. Furthermore, the AlamarBlue assay, which distinguishes living cells by converting resazurin to resorufin and emitting bright red fluorescence (18), has revealed that NP started to kill *A. fumigatus* vegetative cells within 30 minutes and eliminated live cells at 2 hours, comparable to those of 1% Triton buffer exposure (Fig. 7d). Subsequently, samples from the AlamarBlue assay were plated on PDA and no fungal cell proliferations were observed following treatment with NP for 1 hour or longer (Fig. 7e).

### Genome-wide expression response and enrichment analysis of NP-treated *A. fumigatus*

**Fig 8:**
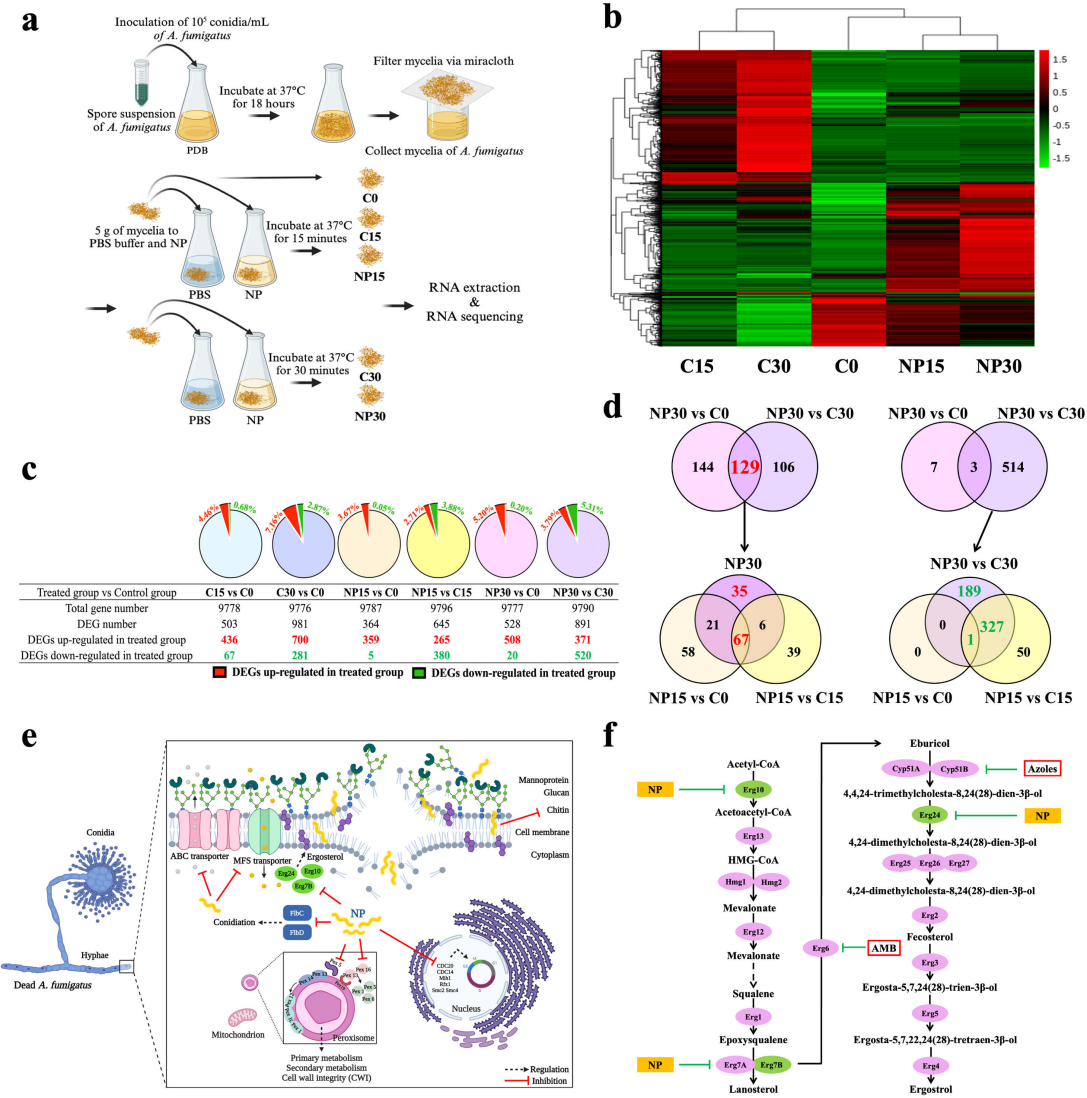
Transcriptomic response of *A. fumigatus* to NP treatment. (a) Schematic representation of the experimental design for treating *A. fumigatus* AF293 vegetative cells with NP for RNA-seq sample collection. (b) Heatmap results from FPKM cluster analysis, clustered using the log2(FPKM + 1) value. Red indicates genes with high expression levels, and green indicates genes with low expression levels. (c) Summary of DEGs in *A. fumigatus* obtained from different treated groups; C15 vs C0, C30 vs C0, NP15 vs C0, NP15 vs C15, NP30 vs C0, and NP30 vs C30. (e) Schematic representation illustrating the transcriptomic responses of *A. fumigatus* exposed to NP. (f) Ergosterol biosynthetic pathway of *A. fumigatus* and downregulated genes affected by NP. RNA-seq was performed in triplicate (*n* = 3).

To understand how *A. fumigatus* cells respond to NP, RNA-seq analysis was conducted by comparing *A. fumigatus* 293 vegetative cells with and without NP treatment, as outlined in Fig. 8a. The heat map generated from FPKM cluster analysis based on RNA-seq data revealed distinct gene expression patterns between the control and NP-treated groups (Fig. 8b). Approximately 2.71%–5.20% of DEGs were upregulated, and 0.05%–5.31% were downregulated in the NP-treated group compared to the control group (Fig. 8c). Core upregulated DEGs were identified, with 129 genes upregulated in the NP30 group compared to both the C0 and C30 groups, and 67 genes common among NP15 vs C0, NP15 vs NP15, and NP30 group, with 35 genes exclusive to the NP30 group (left, Fig. 8d). The core upregulated 67 and 35 DEGs are listed in Tables S1 and S2, respectively. The core downregulated genes included only three genes shared by the NP30 group and both the C0 and C30 groups, while 10 genes were downregulated in NP30 vs C0, and 517 genes in NP30 vs C30. Out of the 517 genes, *flbD* which encodes a MYB family developmental regulator (19) is the sole downregulated DEG consistently identified among the NP-treated groups (Table S3). In addition, 328 genes were downregulated in both NP30 vs C30 and NP15 vs C15 (right, Fig. 8d). Core downregulated DEGs in *A. fumigatus* upon NP exposure are listed in Table S4, with additional 189 downregulated DEGs listed in Table S5.

The analysis of NP’s impact on *A. fumigatus* revealed significant insights into its mechanism of action and specific effects. Table 1 and Fig. 8e present a breakdown of functional categories in *A. fumigatus*, encompassing ergosterol biosynthesis, cell wall maintenance, asexual development, cell stress, cell cycle/division, transport proteins, and DNA repair. The impairment of these critical cellular processes may contribute to the overall reduction in fungal growth and reproduction, aligning with the antifungal activity observed for NP.

**TABLE 1:** Functional classifications of DEGs in *A. fumigatus* on exposure to NP

Gene ID	Gene name	Description	log2 fold change
Ergosterol biosynthesis			
AFUA_1g11230	*hmg2*	HMG-CoA reductase	1.08
AFUA_6G14200	*erg10*	Putative acetyl-CoA acetyltransferase	−2.35
AFUA_4G12040	*erg7B*	Putative lanosterol synthase	−1.66
AFUA_1G03150	*erg24*	Putative C-14 sterol reductase	−1.61
AFUA_1G02570	*erdS*	Ergosteryl-3beta-O-aspartate synthase	−1.34
Cell wall maintenance/remodeling			
AFUA_7G08490		Putative class V chitinase	3.85
AFUA_1G17250	*rodB*	Conidial hydrophobin	3.38
AFUA_3G00610		Putative glucan 1,4-alpha-glucosidase	2.64
AFUA_5G10520		Alpha-1,2-mannosidase family protein	2.32
AFUA_2G00930		Putative xylosidase/glycosyl hydrolase	2.31
AFUA_2G01860		Integral membrane protein	1.56
AFUA_6G02845	*EutQ*	Putative ethanolamine utilization protein	1.45
AFUA_1G05360		CAIB/BAIF family enzyme	1.31
AFUA_3G03060	*aspf34*	cell wall protein PhiA, Allergen	−3.17
AFUA_6G09310		Putative class V chitinase	−2.48
AFUA_4G04180	*chsB*	Putative class II chitin synthase	−1.91
AFUA_6G03600	*Pth11*	Putative integral membrane protein	−1.64
AFUA_5G06670		Integral membrane protein	−1.55
AFUA_4G03240	*Mp1*	Cell wall serine-threonine-rich galactomannoprotein	−1.41
AFUA_4G11720		Putative phosphatidyl synthase	−1.18
AFUA_4G02970	*Aqy1* *TMEM16*	Putative plasma membrane channel protein, dual function calcium channel/ scramblase protein	−1.12
AFUA_3G12330		Putative phosphatidyl synthase	−1.06
Asexual development			
AFUA_1G17250	*rodB*	Conidial hydrophobin	3.38
AFUA_1G03210	*flbD*	MYB family conidiophore development protein	−2.27
AFUA_2G13770	*flbC*	Putative C2H2 zinc finger transcription factor	−2.25
AFUA_5G06410	*AmdA*	Putative C2H2 transcription factor	−2.04
AFUA_2G08470	*bud4*	GTP binding protein	−1.81
AFUA_3G11250	*ace2*	Conidiophore development regulator	−1.08
AFUA_2G07550	*Ark1*	Putative serine/threonine protein kinase	−1.07
Cell stress			
AFUA_8G00100		Putative aspartate-tRNA ligase	3.20
AFUA_8G06020		Glutamate decarboxylase	2.86
AFUA_3G02000		Putative C6 transcription factor Ctf1B-like	2.32
AFUA_5G09100	*mpkC*	MAP kinase	2.19
AFUA_6G07040	*atg5*	Putative autophagy protein	1.75
AFUA_2G00910		Pfs, NB-ARC, and TPR domain protein	1.65
AFUA_6G07050		Putative Na+/H + antiporter	1.53
AFUA_4G03190	*tpsC*	Putative alpha, alpha-trehalose-phosphate synthase subunit	1.43
AFUA_7G08320		Putative heat shock transcription factor	1.17
AFUA_4G01350	*gprK*	G-protein coupled receptor	−2.09
AFUA_6G13570		Putative cytochrome c peroxidase	−2.03
AFUA_2G09850		Putative oxidoreductase, 2-nitropropane dioxygenase family	−2.02
AFUA_3G09640		Putative cAMP-independent regulatory protein	−1.94
AFUA_5G14060	*rho4*	Putative Rho-type GTPase	−1.94
AFUA_7G02570	*TinC*	NIMA-interacting protein	−1.52
AFUA_4G00860	*dprA*	Dehydrin-like protein, cell surface protein	−1.51
AFUA_1G17370	*scf1*	Putative heat shock protein	−1.35
AFUA_6G12180	*dprB*	Dehydrin-like protein	−1.28
AFUA_8G04920		Late embryogenesis abundant (LEA) domain protein	−1.22
AFUA_7G04520	*dprC*	Dehydrin-like protein	−1.08
Cell cycle/division			
AFUA_7G01400	*BimC*	Putative kinesin family protein	−2.61
AFUA_2G16260	*Ase1*	Putative microtubule-associated protein	−1.96
AFUA_8G05680		Putative serine/threonine protein kinase	−1.80
AFUA_3G12820		Kinesin family protein	−1.62
AFUA_3G10250	*Cdc15*	Putative cell division control protein	−1.61
AFUA_2G04140	*Spc105*	Putative chromosome segregation protein	−1.48
AFUA_3G12250	*cdcA*	Putative tyrosine-protein phosphatase CDC14	−1.45
AFUA_3G10180		HEC/Ndc80p family protein	−1.45
AFUA_6G02670	*nimA*	Putative cell-cycle regulated serine/threonine protein kinase, G2-specific protein kinase	−1.24
AFUA_1G14730	*Cdc20*	Putative cell division cycle protein	−1.24
AFUA_6G12980		Putative spindle pole body component	−1.22
AFUA_6G08200	*nimT* *Mih1*	Putative M-phase inducer phosphatase Putative cell cycle control protein tyrosine phosphatase	−1.15
AFUA_6G08530	*Src1*	Putative sister chromatid separation protein	−1.12
AFUA_2G02170	*smc4*	Putative nuclear condensin complex subunit	−1.07
AFUA_2G03150		Kinesin family protein	−1.06
Transport proteins			
AFUA_7G00220		Putative plasma membrane hexose transporter	2.15
AFUA_3G02150		Putative MFS monocarboxylate transporter	2.13
AFUA_6G07050		Putative Na+/H + antiporter	1.53
AFUA_2G09460		Potassium transporter	1.53
AFUA_3G02890		Putative MFS sugar transporter	1.40
AFUA_6G02810		Putative low-affinity copper transporter	1.29
AFUA_6G03690	*ena1*	Putative P-type ATPase sodium pump	−9.06
AFUA_4G01560		Putative MFS myo-inositol transporter	−7.88
AFUA_4G09440		Putative P-type ATPase sodium transporter	−5.85
AFUA_3G01370		Putative MFS transporter	−4.76
AFUA_8G02550		Putative MFS peptide transporter	−4.40
AFUA_8G05710	*mfsA*	Putative MFS sugar transporter	−3.94
AFUA_4G00800		Putative monosaccharide transporter	−3.55
AFUA_3G11790		Putative galactose-proton symport	−3.06
AFUA_8G06870		Putative MFS sugar transporter	−2.99
AFUA_8G02010		Putative MFS sugar transporter	−2.95
AFUA_1G16690		Putative MFS sugar transporter	−2.75
AFUA_4G02870		Putative 2-ketogluconate transporter	−2.40
AFUA_1G04780	*pxa1*	Putative peroxisomal ABC transporter	−2.38
AFUA_1G14330	*abcC*	Putative ABC transporter, Azole transporter	−2.35
AFUA_7G00950		Putative MFS monosaccharide transporter	−2.25
AFUA_2G12500		Putative MFS multidrug transporter	−2.25
AFUA_3G01840		Putative MFS transporter	−2.25
AFUA_6G03060		Putative MFS monosaccharide transporter	−2.14
AFUA_4G03750		Putative phthalate transporter	−2.05
AFUA_6G03720		Putative MFS allantoate transporter	−2.02
AFUA_4G00150		Putative MFS maltose transporter	−2.01
AFUA_5G02840		Putative MFS sugar transporter	−1.88
AFUA_7G06390	*MalP*	Putative MFS alpha-glucoside transporter, MFS maltose permease	−1.74
AFUA_7G00780		Putative MFS monocarboxylate transporter	−1.73
AFUA_6G00130		Putative MFS transporter	−1.57
AFUA_8G00720		Putative amino acid transporter	−1.48
AFUA_2G12550		Putative MFS multidrug transporter	−1.43
AFUA_4G09150		Putative ABC multidrug transporter	−1.38
AFUA_5G11980		MFS efflux transporter	−1.33
AFUA_6G00440		Putative cation diffusion facilitator	−1.27
AFUA_2G14590		Putative MFS monosaccharide transporter	−1.16
AFUA_8G06760		Putative MFS transporter	−1.09
DAN repair			
AFUA_3G12280	*Translin*	Putative recombination hotspot-binding protein	1.43
AFUA_1G07000	*Rad26*	Putative DNA repair protein	1.13
AFUA_2G16260	*Ase1*	Putative microtubule-associated protein	−1.96
AFUA_5G06120	*Rfx1*	Putative DNA damage and replication checkpoint protein	−1.36
AFUA_3G14260		Putative mismatched base pair and cruciform DNA recognition protein	−1.27
AFUA_4G04390	*tof1*	Putative topoisomerase 1-associated factor 1, DNA repair protein	−1.14

In particular, ergosterol, a vital component in the fungal cell membrane, plays a crucial role in development and growth and is a primary target for existing antifungal drugs (20). The downregulation observed in genes such as *ERG10*, *ERG7B*, *ERG24*, and *ERDS* within the ergosterol biosynthesis pathway indicated that NP may impede the production of key intermediates like acetoacetyl-CoA, lanosterol, and 4,24-dimethylcholesta-8,24,(28)-dien-3β-ol. This parallels the effects seen with antibiotics like AMB and azole antifungals, which suppress genes such as *ERG6* (21) and *ERG11* (CYP51A/B) (22), respectively, targeting ergosterol biosynthesis. This indicates a potential blockade in the ergosterol biosynthesis pathway induced by NP, providing insights into its antifungal mechanism (Fig. 8f).

### Gene regulatory network of NP-treated *A. fumigatus*

**Fig 9:**
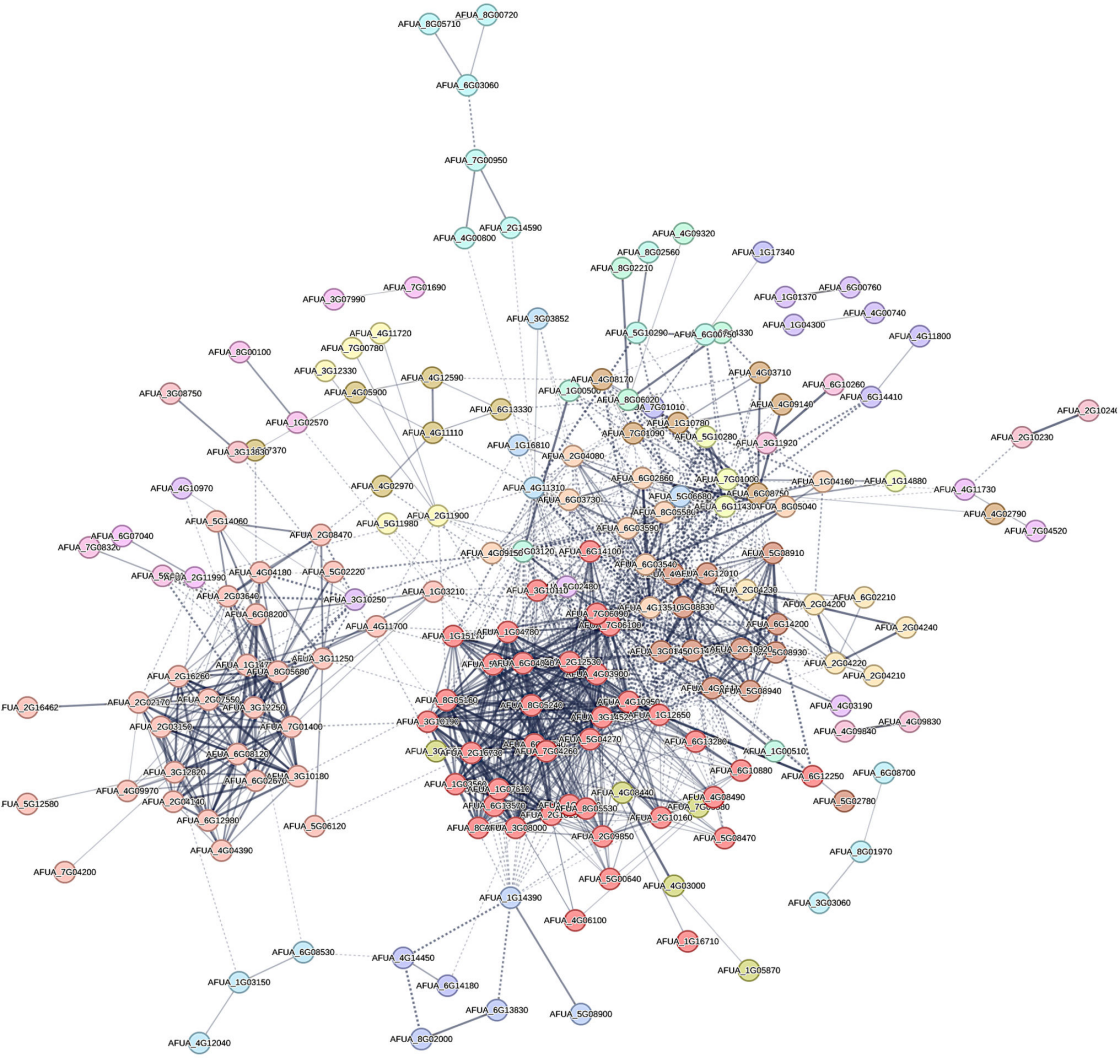
PPI in the degree-sorted network of 328 downregulated DEGs and 67 upregulated DEGs. PPI was constructed in the STRING database and depicts the interactions among the shared DEGs with a confidence score of 0.4 with at least three nodes connected. The node colors represent MCL clustering with granularity parameters as 4.

The regulatory effects of NP on *A. fumigatus* were investigated through an extensive network analysis, integrating RNA-seq data with the protein-protein interaction (PPI) database. The resulting network, comprising 22 clusters, contained 395 nodes and 1,113 edges, derived from 328 downregulated DEGs and 67 upregulated DEGs (Fig. 9), with their functional enrichment detailed in Table S6. Cluster 1, comprising 29 nodes and 258 edges, emerged as a focal point enriched in pathways related to unsaturated fatty acid biosynthesis, beta-alanine metabolism, fatty acid degradation, propanoate metabolism, and peroxisome function. Peroxisomes, known for their involvement in various metabolic processes including fatty acid breakdown and detoxification through hydrogen peroxide generation (23, 24), were particularly prominent. These findings suggest that NP may exert its antifungal properties by affecting multiple enzymes involved in critical metabolic processes, offering valuable insights into its multifaceted regulatory effects on *A. fumigatus*.

## DISCUSSION AND CONCLUSIONS

Antibiotic resistance is a pressing global concern, echoing Alexander Fleming’s early warnings about antibiotic misuse. The emergence of penicillin-resistant *E. coli* in 1940 highlighted Fleming’s concerns (25). A meta-analysis pooling data from various studies on antibiotic resistance in pathogens isolated from food revealed alarming statistics. Over 11% of foodborne pathogens showed antimicrobial resistance, with multi-drug resistant (MDR) pathogens exceeding 36% across all food categories. In addition, biofilm formation capacity among foodborne pathogens, contributing to antimicrobial resistance (AMR), was observed, with *S. aureus* exhibiting the highest propensity (26). Despite this, there is no comprehensive international surveillance system for AMR. However, reports suggest over 2 million AMR infections occur annually in the United States, leading to around 29,000 deaths. Similarly, in Europe, over 33,000 deaths are linked to AMR infections (27).

ESKAPE pathogens, part of AMR pathogens, are critical targets for new antibiotic development due to the decreasing efficacy of existing antibiotics, signaling a worrying trend (5), and necessitating alternative strategies. Unlike the diversity of antibiotics for bacterial infections, antifungal options are limited primarily to azoles, echinocandins, and polyenes like amphotericin B (6). Fungal infections cause about 1.5 million deaths, with over 90% attributed to *Cryptococcus*, *Candida*, *Aspergillus*, and *Pneumocystis* species, known for causing invasive fungal infections (28).

Innovative antimicrobial approaches such as engineered antibodies, antibiotic enhancers, and CRISPR/Cas-enabled genome editing are emerging, albeit with significant costs and time requirements (29, 30). In addition, there is a growing consumer preference for minimally processed, “clean label” products, driving interest in natural antimicrobials perceived as safer and environmentally friendly (31). Consequently, fermentation technology introduces “postbiotics,” defined as “preparations of inanimate microorganisms and/or their components that confer a health benefit on the host,” holding significant potential in antimicrobial therapy (32).

We anticipate NP’s stability and potent antimicrobial activity to make it a formidable broad-spectrum agent against both AMR bacterial and fungal pathogens. NP can eradicate MRSA within 6 hours, *L. monocytogenes* within 3 hours, *E. coli* O157:H7 within 24 hours, and *S. typhimurium* within 36 hours, achieving a 3-log reduction in bacterial counts. It also inhibits biofilm-producing ESKAPE strains like *K. pneumoniae* and *P. aeruginosa*, even in nutrient-rich environments. NP impedes spore germination and halts the growth of *A. fumigatus* vegetative cells, including clinically isolated strains CEA-10 and CEA-17. In addition, it shows fungistatic effects against *C. albicans* for the first 24 hours, with some cidal activity observed after 24 hours. NP can also target food spoilage-causing *Penicillium* species such as *P. roqueforti*, *P. chrysogenum*, and *P. expansum*. Various NP application methods can be envisioned, including soaking/misting susceptible food products with NP, employing NP for medical purposes in treating skin infections or incorporating a lyophilized NP tablet into different food items.

Treating fungal infections has been challenging due to the similarity between fungi and human hosts, both being eukaryotic organisms. However, RNA-seq analysis of *A. fumigatus* revealed downregulation of genes associated with ATP-binding cassette (ABC) superfamily and major facilitator superfamily (MFS) transporters, potentially reducing NP efflux (33) and enhancing its antifungal efficacy. Moreover, examination of DEGs associated with cell wall maintenance and remodeling showed NP’s multifaceted impact on fungal physiology. This included the upregulation of genes like conidial hydrophobin B (*RodB*), pivotal for constructing the outer cell wall of conidia (34), and chitinase activity genes essential for processes such as conidial germination, mycelial formation, and cell wall expansion (21, 35). In addition, the downregulation of genes associated with conidiation, such as the MYB family conidiophore development protein (*flbD*) and C_2_H_2_ zinc finger transcription factor (*flbC*), indicated a potential impact on the formation of asexual spores (19). The modulation of genes encoding integral membrane proteins, cell wall proteins, and chitinase indicated a concerted effort by the fungus to maintain cell wall integrity under NP exposure, possibly as a survival strategy. Moreover, although NP likely affects membrane permeability, our findings suggest that its impact extends beyond mere membrane disruption. Unlike detergents like SDS, which cause non-specific membrane damage, NP appears to interact with specific cellular targets, leading to complex transcriptional responses. This is evidenced by the diverse pathways affected, indicating that NP induces a broader range of cellular responses. Thus, the transcriptomic changes observed are likely reflective of a more intricate interaction between NP and fungal cellular pathways, rather than being solely attributable to cell death or membrane disruption.

In conclusion, this study introduces a food-grade broad-spectrum antimicrobial fermentate applicable to both food sources and medicine for pathogen control, enhancing antibiotic effectiveness, and addressing antimicrobial resistance. NP serves as a promising “clean label” solution for diverse applications across pharmaceutical and food industries, paving the way for safeguarding global health and food safety.

## MATERIALS AND METHODS

### Fungi and culture conditions

For NP production, the mold strain *A. oryzae* NRRL 3483 was used. Various strains of *A. fumigatus* (AF293, F16216, F11628, CEA-10, and CEA-17) and *Penicillium* species (*P. roqueforti*, *P. chrysogenum*, *P. expansum* Pe21, and *P. expansum* Pe19) were employed to assess NP’s antifungal activity. For inoculum preparation, *A. oryzae* was cultured on potato dextrose agar (PDA) for 4 days at 30°C, while *A. fumigatus* strains and *Penicillium* species were cultured at 37°C for 3 days and at 25°C for 5 days, respectively. A 0.1% Tween-80 solution was used to harvest asexual spores (conidia) from the PDA, followed by enumeration using a hemocytometer. To assess the antifungal activity of *A. fumigatus* vegetative cells, a spore suspension (10^6^ conidia/mL) was inoculated into potato dextrose broth (PDB) and incubated at 37°C for 18–24 hours with shaking at 220 rpm. After incubation, *A. fumigatus* mycelia were harvested by filtration through four layers of Miracloth (MilliporeSigma, USA). *C. albicans* SC5314 was used to evaluate NP’s anti-yeast activity, with single colonies cultured on yeast peptone dextrose (YPD) agar plates at 30°C overnight.

### Bacteria and culture conditions

The antibacterial efficacy of NP was evaluated against a spectrum of bacterial strains including *L. monocytogenes* 10403S, *S. aureus* S6, *E. coli* K12 FRIK 2637 (non-pathogenic strain), *E. coli* O157:H7 FRIK 47, and *S. enterica typhimurium* S9. In addition, testing extended to highly virulent and antibiotic-resistant ESKAPE strains, comprising two *S*. *aureus* strains (MW2, 33593), eight methicillin-resistant *S. aureus* (MRSA) strains (OC11521, two isolates of OC4222, three isolates of OC8530, 04-045, OC17042), two *K. pneumoniae* strains (81-1269A, BAA-2146), three *P. aeruginosa* strains (2638, 3060, 65), and two *E. coli* strains (2671, 1-894-1).

Bacterial suspensions were prepared following established protocols with slight adjustments (36, 37). Isolated colonies were selected from culture plates and transferred into 4–5 mL of tryptic soy broth (TSB) liquid medium for all bacterial strains. Cultures were then incubated at 37°C for 2–6 hours to reach the log phase of growth, as determined by the 0.5 McFarland standard, corresponding to approximately 1.0 × 10^8^ CFU/mL when the optical density fell between 0.08 and 0.1. Prior to the experiment, a 1:20 dilution was made by mixing 2.0 mL of the original suspension with 38 mL of PBS buffer, followed by a 1:10 dilution by adding 0.01 mL to each well to achieve a final concentration of approximately 5.0 × 10^5^ CFU/mL.

### Preparation of media for NP determination

To identify the optimal medium for NP evaluation, several media with distinct compositions were prepared. These included Tryptic Soy Broth (TSB), Yeast Peptone Dextrose (YPD), De Man, Rogosa & Sharpe (MRS), Luria-Bertani (LB), M17, Mueller Hinton (MH), Brain Heart Infusion (BHI), Czapek (CZ), Potato Dextrose Broth (PDB), Malt Extract Broth (MEB), and Minimal Medium (MM). The composition of each medium (in grams per liter of distilled water) is detailed in Table S7. All liquid media were autoclaved at 121°C and 15 PSIG for 20 minutes to ensure sterilization.

### Preparation of NP

The full-strength culture medium for NP (TSB) was prepared by dissolving all ingredients (refer to Table S8) and subsequently sterilized under high pressure (15 PSIG for 20 minutes at 121°C). To initiate NP production, *A. oryzae* NRRL 3483 was inoculated at a final concentration of 5 × 10^5^ conidia/mL into 250 mL Erlenmeyer flasks containing 150 mL of NP culture medium. The inoculated flasks were incubated for 6 days at 30°C with shaking at 220 rpm. Post-incubation, mycelia were separated from the culture broth through filtration using four layers of Miracloth (MilliporeSigma, USA). Subsequently, “NP,” a sterile, cell-free culture fermentate, was obtained by filtration through a 0.22-µm polyethersulfone (PES) membrane filter unit (Thermo Fisher Scientific, USA). The schematic representation of the NP production process is illustrated in Fig. 1a.

### Preparation and assessment of NP-EAE)

#### Preparation of NP-EAE *via* liquid-liquid extraction

The antimicrobial activity of NP extracts, obtained *via* liquid-liquid extraction using different organic solvents, was assessed. Details regarding the solvents utilized are provided in Table S9, while the procedure for producing NP-EAE is outlined in Fig. 1b. Ethyl acetate extracts exhibited potent antibacterial activity among the solvents tested, therefore referred to as “NP-EAE.”

### Preparation of NP-EAE *via* liquid-liquid extraction

Antimicrobial activity screening of NP extracts was carried out using liquid-liquid extraction with various organic solvents (listed in Table S9). In 50 mL conical tubes, 20 mL of NP was mixed with an equal volume of the respective organic solvent (vol/vol) and incubated overnight at 25°C with shaking at 150 rpm. After centrifugation at 5,000 g, the organic layer was transferred to a new tube. Extracts were dried under gentle airflow, reconstituted in 1 mL of methanol, and filtered using a sterile 0.45-µm PES membrane filter unit (Thermo Fisher Scientific, USA). The resulting methanol solution was then tested for antimicrobial activity, with methanol serving as the negative control. Among the solvents tested, ethyl acetate extracts exhibited potent antibacterial activity. Subsequent experiments focused on the ethyl acetate extract, referred to as “NP-EAE.” A concentrated ethyl acetate extract equivalent to 20 times the NP fermentate, labeled as “20×,” was obtained through a subsequent ethyl acetate extraction on 1 mL of the methanol solution. This process was repeated three times, resulting in “60×” NP-EAE.

### Agar diffusion disk test assay

Agar diffusion disk testing, with slight modifications, was conducted to assess the antibacterial and antifungal activities of NP-EAE, following a previously described protocol based on CLSI guidelines (36–38). For antibacterial activity assessment, 100 µL of NP-EAE was applied to 6 mm sterile paper disks, while for antifungal activity evaluation, 300 µL of NP-EAE was applied to 13 mm sterile paper disks. The paper disks were dried under the fume hood and placed on the surface of the TSA plates for antibacterial activity and PDA plates for antifungal activity. The plates were inverted and incubated at 37°C for 18–22 hours to cultivate bacteria. As for *Penicillium sp*. and *Aspergillus sp*., they were incubated at 25-37°C, for 4–6 days. Positive controls for bacterial testing included disks loaded with 5 µg ofloxacin (OFX 5; OXOID Ltd, USA), 30 µg of cefoxitin (FOX 30; OXOID Ltd, USA), and 10 µg Ampicillin (Sigma-Aldrich, USA). Positive controls for antifungal activity included amphotericin B (AMB; Sigma-Aldrich, USA) at a final concentration of 4 µg/mL.

### Preparation and assessment of NP-F

#### Preparation of NP-F

In preparation of NP-F for evaluating both antibacterial and antifungal activities, NP solutions were prepared by diluting NP in distilled water at varying concentrations (i.e., 100%, 50%, 25%, 12.5%, 6%, and 0%) described in Fig. 1c. These solutions were then used as solvents to produce 22 g/L Mueller Hinton Broth (MHB) contained 2.0 g beef extract, 17.5 g acid hydrolysate of casein and 1.5 g starch for antibacterial testing (37), and the antifungal activity test broth having 10.4 g/L RPMI 1640 powder supplemented with 2% glucose and 3-(N-morpholino) propanesulfonic acid (MOPS) at a final concentration of 0.165 mol/L (pH 7.0) (38) or 39 g/L PDA solid medium.

### Time-kill assay in NP-F liquid medium

A modified version of the broth microdilution method was conducted to evaluate the antibacterial and antifungal properties of NP-F, following a previously described protocol based on CLSI guidelines with modifications (36–38). The original nutrient medium served as a negative control.

### Fungal growth on NP-F solid medium

Briefly, 10 µL of spore suspensions from *Penicillium* species and five strains *A. fumigatus* were spot plated onto NP-F (PDA). The spore concentrations varied from 10^1^ to 10^4^ spores per spot. In addition, a control group with 0 spores per spot was inoculated with 10 µL of distilled water, as the negative control.

### Membrane permeability assay

#### Membrane integrity in bacterial cells using fluorescent dyes

Using an electron fluorescence spectrometer (Tecan, Switzerland), inner and outer membrane integrity assays were conducted with specific fluorescent dyes, as depicted in Fig. S7. The influx assay utilized SYTOX Green (Invitrogen, USA) to evaluate the permeability of *S. aureus* S6’s inner membrane, following established protocols with slight modification (39). SYTOX Green’s excitation and emission wavelengths were measured at 480 and 522 nm, respectively, with 20 µL of 5 µM dye added to the wells. For assessing the permeability of *E. coli* K12 FRIK 2637’s outer membrane, an N-phenyl-1-napthylamine (NPN) assay (Sigma-Aldrich, USA) was employed. Excitation and emission wavelengths were set at 350 and 429 nm, respectively, with samples mixed with 20 µL of NPN solution (final concentration: 10 µM) as described previously (40).

To prepare NP-EAE samples, 100 µL of NP-EAE (reconstituted in methanol) was transferred into a sterile 96-well plate and dried under a fume hood. Subsequently, 160 µL of PBS buffer was added to the wells. Following this, 20 µL of bacterial cultures (~10^5^ CFU/mL, obtained *via* serial dilution of overnight bacterial cultures) was added to each well and incubated them at 37°C for 6 hours.

### Release of 260 nm absorbing materials from bacterial cytoplasm

The release of materials such as nucleic acids from the cytoplasm was detected by measuring absorbance at 260 nm after 3-hour treatments following the procedure outlined previously (41).

### Flow cytometry

To corroborate the observations on cell membrane permeability elucidated earlier, flow cytometry (BD Accuri, USA) was employed. After a 6-hour incubation of bacterial cells and fungal spores with NP-EAE and control, SYTO-9 and propidium iodide (PI) (Invitrogen, USA) were added at concentrations of 7.5 µM and 30 µM, respectively, according to previously outlined protocols (42). Fluorescence intensities were measured using a green fluorescence (525/50 nm) bandpass filter for SYTO 9 and a red fluorescence (610/20 nm) bandpass filter for PI.

### Alamarblue HS cell viability assay

The impact of NP on the growth of *A. fumigatus* mycelia (actively growing vegetative cells) was assessed by quantifying cell viability and proliferation using the Alamarblue HS Cell Viability assay (Thermo Fisher Scientific, USA) following the manufacturer’s protocol. Five grams of harvested mycelia, which were cultivated for 18 hours in PDB, subjected to four different solutions: NP-producing fungus-free medium (negative control), PBS buffer (negative control), NP, and 1% Triton buffer (positive control), all at a volume of 25 mL. After 0, 0.5, 1, 2, 3, 4, 5, and 6 hours of incubation, 10 µL of Alamarblue reagent was added to 90 µL of mycelia suspended in PDB liquid medium. Fluorescence was measured using a fluorescence excitation wavelength of 570 nm (with an excitation range of 540–570 nm) and an emission wavelength of 600 nm (with an emission range of 580–610 nm). The percentage reduction of Alamarblue reagent was determined at predefined time intervals using Eq. 1 according to the manufacturer’s instructions.

### Mammalian cell cytotoxicity

NP along with controls was lyophilized using a freeze dryer (Yamato, USA) in preparation for cell cytotoxicity test assay. A mammalian cell cytotoxicity assay was conducted by the Cancer Pharmacology Lab at the University of Wisconsin-Madison. The lyophilized samples were reconstituted in sterile molecular biology-grade water and were dispensed onto white 384-well plates (Corning 3765, Thermo Fisher, USA) at final concentrations ranging from 10 to 1,000 µg/mL using an Echo 650 acoustic liquid handler (Beckman Coulter, USA). MCF7 cells (breast cancer cell line) were then exposed to the samples for 72 hours, and toxicity was assessed using the CellTiter-GLO reagent (Promega, USA) following the manufacturer’s protocol. Doxorubicin, a chemotherapy drug, served as a positive control at a concentration of 2.5 µM.

### Genome-wide expression studies

#### Preparation of RNA samples

Samples for RNA sequencing analysis were treated as detailed in Fig. 8a. Following treatments at distinct time points, total RNAs from individual samples were extracted using RNeasy Kits (Qiagen, Germany) following the manufacturer’s instructions.

### RNA sequencing analysis

The total RNA samples underwent quality assessment, library preparation, and mRNA sequencing by Novogene company (Beijing, China). The quality of total RNA was validated as described (43). The genome and gene annotations for *A. fumigatus* AF293 were from the National Center for Biotechnology Information (NCBI) website (https://www.ncbi.nlm.nih.gov/; GCA_002234985.1). The differentially expressed genes (DEG) level was processed using DESeq2 (version 1.20.0). Genes with an adjusted *P*-value  <  0.05 and |log2(FoldChange)|  > 0 were considered as the threshold for significant differential expression.

### Protein-protein interaction analysis

A network analysis was performed by integrating the results of RNA-seq with the protein-protein interaction (PPI) database. Subsequently, known PPI information of *A. fumigatus* AF293 was obtained from the gene network database STRING (version 11.5) (44). This network illustrates interactions among the shared DEGs with a confidence score of 0.4 and at least three connected nodes. Each cluster within the network was subjected to KEGG pathway enrichment analysis to identify functional enrichments (45).

### Appendices


(1) $$\%\text{ Alamarblue Reduction }=\frac{(\text{ Eoxi }600\times A570)-(\text{ Eoxi }570\times A600)\times 100}{\left(\text{ Ered }570\times C600)-(\text{ Ered }600\times C570\right)}$$

where;

*E_oxi600_* & *E_oxi570_*: E of oxidized Alamarblue at 600 nm (117216) and 570 nm (80586),

*E_red600_* & *E_red570_*: E of reduced Alamarblue at 600 nm (14652) and 570 nm (155677),

*A_600_* & *A_570_*: Absorbance of test wells at 600 nm and 570 nm, and

*C_600_* & *C_570_*: Absorbance of negative control well (media plus alamarblue but no cells) at 600 nm and 570 nm

## SUPPLEMENTAL MATERIAL

Supplemental figures spectrum.01854-24-s0001.docx Fig. S1 to S7.

Supplemental tables spectrum.01854-24-s0002.docx Tables S1 to S9.

ASM does not own the copyrights to Supplemental Material that may be linked to, or accessed through, an article. The authors have granted ASM a non-exclusive, world-wide license to publish the Supplemental Material files. Please contact the corresponding author directly for reuse.
