# Supplementary material for: Broad-spectrum antimicrobial activities of a food fermentate of Aspergillus oryzae
Source: Microbiol Spectr. 2024 Oct 22;12(12):e01854-24. doi: 10.1128/spectrum.01854-24 (PMC11619415; doi:10.1128/spectrum.01854-24)
Supplement: Supplemental figures — Fig. S1 to S7. [file spectrum.01854-24-s0001.docx]

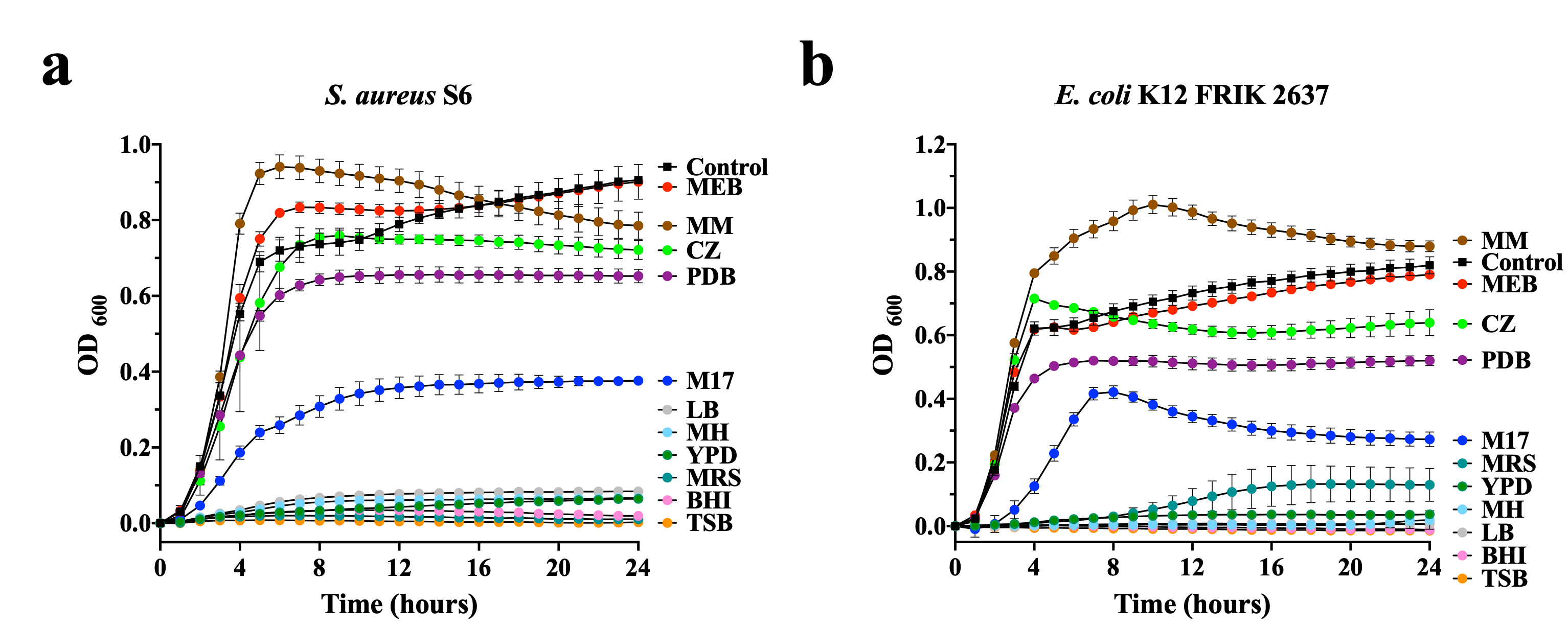


**Figure S1**. **Identification of proprietary media for NP production.** Bacterial growth curves of (a) *S. aureus* S6 and (b) *E. coli* K12 FRIK 2637 in TSB liquid medium supplemented with various fermentates. Optical density at 600 nm (OD600) was measured every hour for 24 hours at 37°C using a Bioscreen C system. The original TSB medium served as the negative control. The media compositions are detailed in Table S7. Experiments were performed in triplicate (n=3).


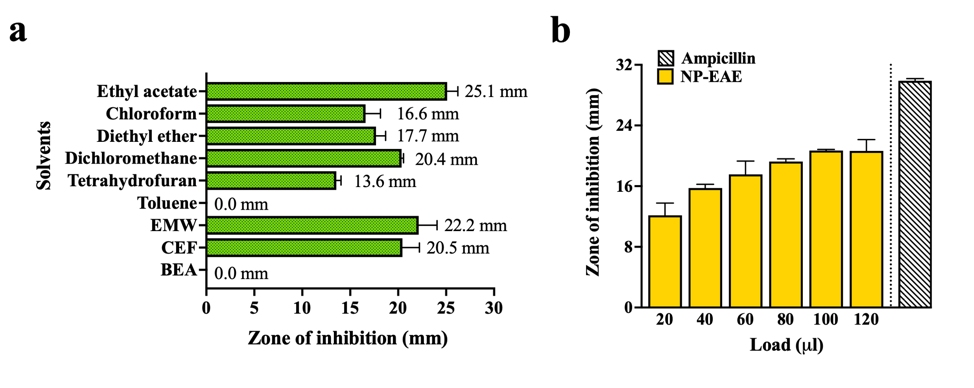


**Figure S2**. **Determining the optimal solvent and the volume of NP-EAE by assessing the zone of inhibition against *S. aureus* S6.** (a) NP extracted with various organic solvents. (b) Different volumes of the NP-EAE. Disks were loaded with 20, 40, 60, 80, 100, or 120 µL of the NP-EAE. A disk loaded with 10 µg of ampicillin was used as the positive control. Experiments were performed in triplicate (n=3).


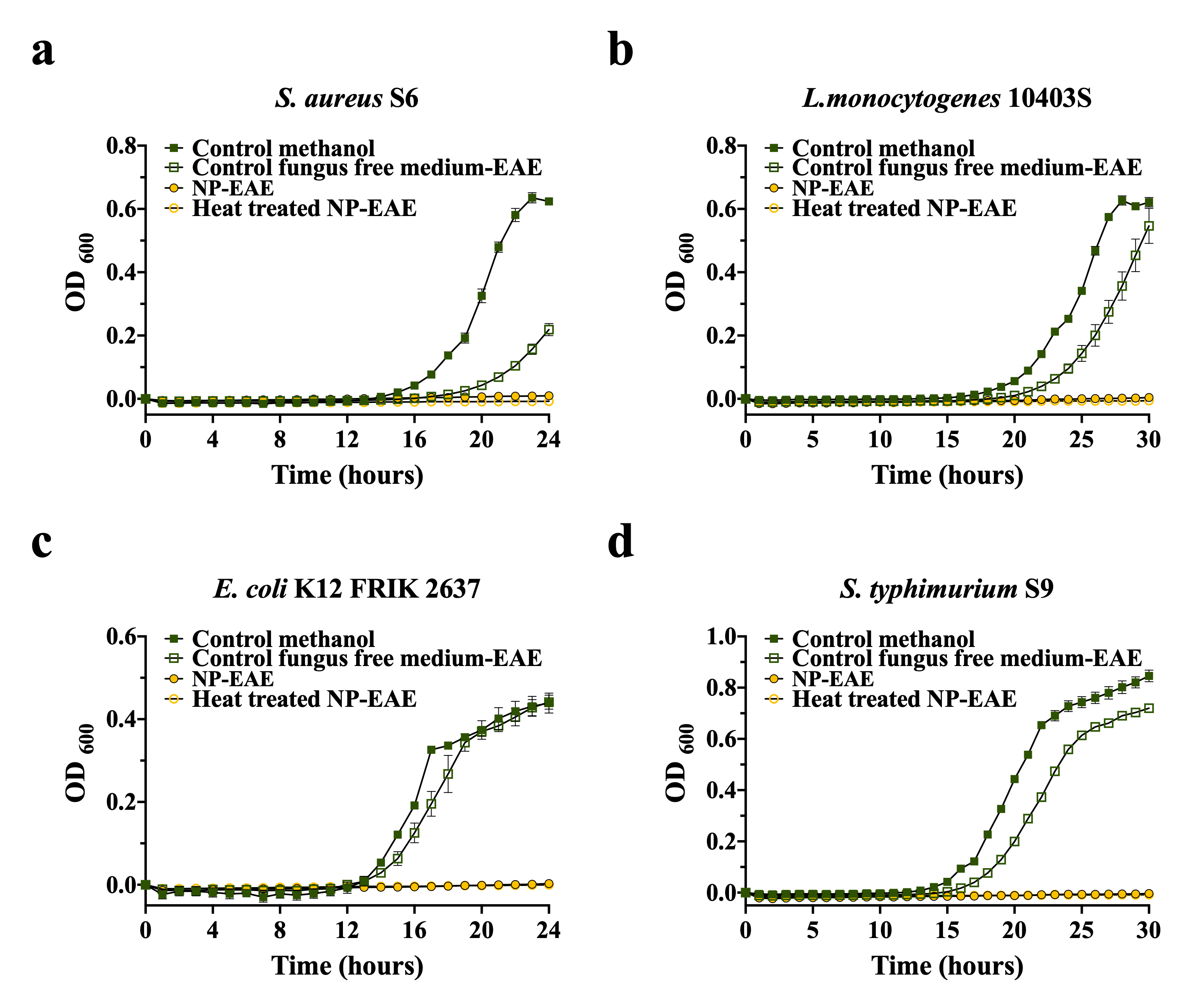


**Figure S3**. **Antibacterial activities of NP-EAE.** Bacterial growth curves of (a) *S. aureus* S6, (b) *L. monocytogenes* 10403S, (c) *E. coli* K12 FRIK 2637, and (d) *S. typhimurium* S9, cultured in the presence of ethyl acetate extract (10x NP equivalent) in TSB liquid medium at 25°C for 24–30 hours. OD600 was recorded hourly using the Bioscreen C system. “Heat-treated NP-EAE” refers to NP subjected to autoclaving (121°C, 15 PSIG) before extraction with ethyl acetate. Negative controls included methanol and ethyl acetate extracts of fungus-free medium used to produce NP.

Experiments were performed in triplicate (n=3)


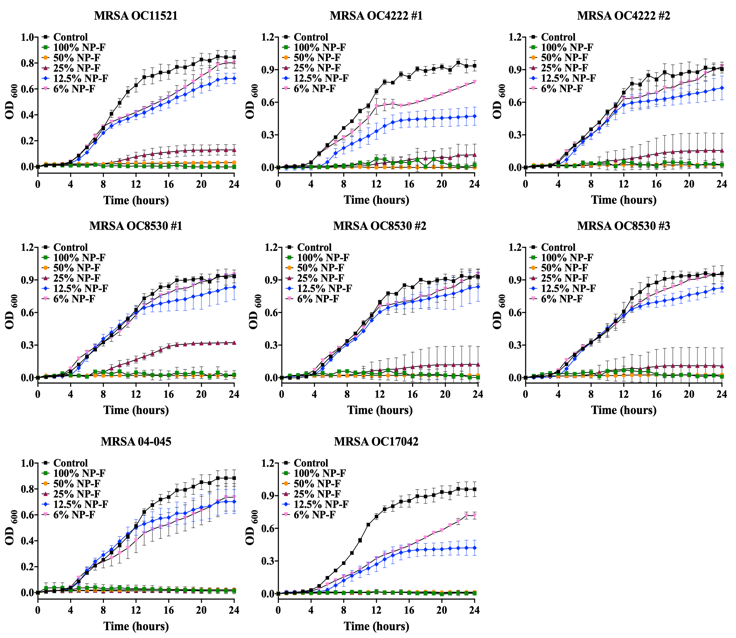


**Figure S4. Antibacterial activities of NP-F against 8 MRSA strain.** Bacterial growth curves of 8 MRSA strains in NP-F (100%, 50%, 25%, 12.5% and 6%) as a solvent at 37°C for 24 hours. Original MHB medium was used as negative control. Experiments were performed in triplicate (n=3).


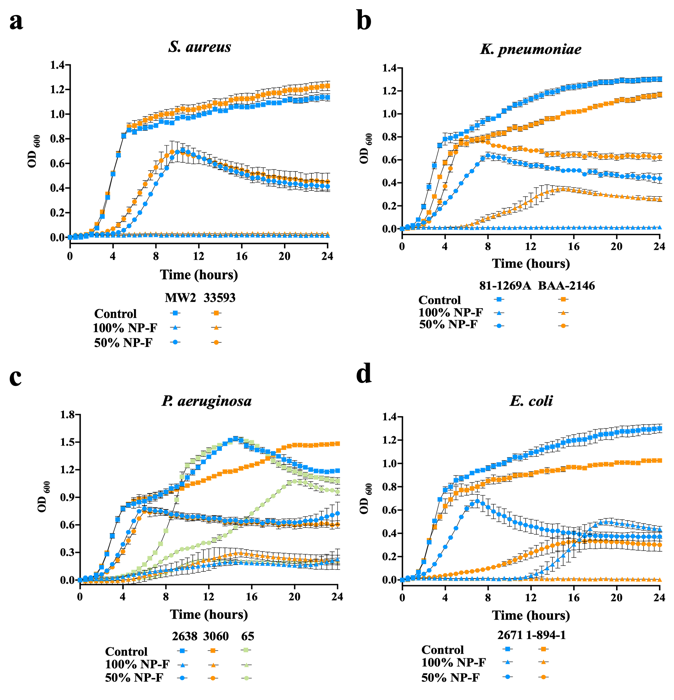


**Figure S5. Antibacterial activities of NP-F against ESKAPE pathogens.** Bacterial growth curves of (a) *S. aureus* MW2 and 33593, (b) *K. pneumoniae* 81-1269A and BAA-2146, (c) *P. aeruginosa* 2638, 3060 and 65, (d) *E. coli* 2671 and 1-894-1 in NP-F (100%, and 50%) as a solvent at 37°C for 24 hours. Original MHB medium was used as negative control. Experiments were performed in triplicate (n=3).

**Figure S6**. **Antifungal activity of the NP-F.** Inhibition of colony growth of (a) three different *Penicillium* species (*P. roqueforti*, *P. chrysogenum*, and *P. expansum*) and (b) five different strains (AF293, F16216, F11628, CEA-10, CEA-17) of *A. fumigatus* in NP-F (PDA) with different various percentage of NP (100%, 50%, 25%, and 10%) as a solvent. *P. roqueforti* and *A. fumigatus* were inoculated at conidial counts of 0, 10, 100, 1,000, and 10,000 per spot and grown at 25°C and 37°C for 3-5 days, respectively. Original PDA solid medium was used as negative control (PDA). Experiments were performed in triplicate (n=3).


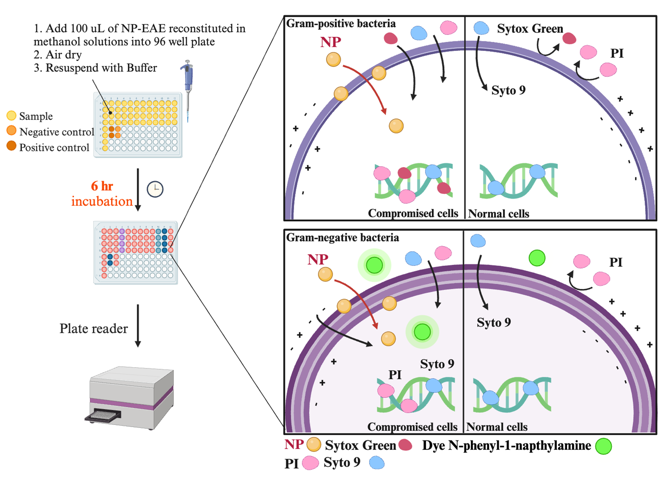


**Figure S7: Experimental procedure to determine NP-EAE’s effects on bacterial membrane.** Created with Biorender.com.
