# Supplementary material for: Broad-spectrum antimicrobial activities of a food fermentate of Aspergillus oryzae
Source: Microbiol Spectr. 2024 Oct 22;12(12):e01854-24. doi: 10.1128/spectrum.01854-24 (PMC11619415; doi:10.1128/spectrum.01854-24)
Supplement: Supplemental tables — Tables S1 to S9. [file spectrum.01854-24-s0002.docx]

**Table S1. Core 67 up-regulated DEGs in *A. fumigatus* on exposure to NP**

| Gene ID | Gene Name | Description | log2 Fold Change |
| --- | --- | --- | --- |
| AFUA_7G08490 |  | Putative class V chitinase | 3.85 |
| AFUA_1G17250 | *rodB* | Conidial hydrophobin | 3.38 |
| AFUA_3G13570 |  | Hypothetical protein | 3.38 |
| AFUA_7G00300 |  | Putative squalene-hopene-cyclase | 3.34 |
| AFUA_8G00100 |  | Putative aspartate-tRNA ligase | 3.20 |
| AFUA_8G00150 |  | Hypothetical protein | 3.15 |
| AFUA_8G06020 |  | Glutamate decarboxylase | 2.86 |
| AFUA_8G00110 |  | Putative oxidoreductase, 2OG-Fe(II) oxygenase family | 2.83 |
| AFUA_1G01440 |  | Hypothetical protein | 2.78 |
| AFUA_7G08480 |  | Putative SWI/SNF family DNA-dependent ATPase | 2.76 |
| AFUA_5G10320 | *GliH* | Putative toxin biosynthesis protein | 2.75 |
| AFUA_2G00920 | *abfII* | Putative extracellular glycosyl hydrolase/cellulase | 2.69 |
| AFUA_3G00610 |  | Putative glucan 1,4-alpha-glucosidase | 2.64 |
| AFUA_3G03140 |  | HET domain protein | 2.59 |
| AFUA_3G13450 |  | Oxidoreductase, short chain dehydrogenase/reductase family superfamily | 2.39 |
| AFUA_3G02000 |  | Putative C6 transcription factor Ctf1B-like | 2.32 |
| AFUA_5G10520 |  | Alpha-1,2-mannosidase family protein | 2.32 |
| AFUA_2G00930 |  | Putative xylosidase/glycosyl hydrolase | 2.31 |
| AFUA_3G02700 |  | Putative C6 transcription factor | 2.23 |
| AFUA_7G07040 |  | Hypothetical protein | 2.20 |
| AFUA_5G09100 | *mpkC* | MAP kinase | 2.19 |
| AFUA_7G00220 |  | Putative plasma membrane hexose transporter | 2.15 |
| AFUA_3G02150 |  | Putative MFS monocarboxylate transporter | 2.13 |
| AFUA_3G03150 |  | Hypothetical protein | 2.12 |
| AFUA_1G13980 |  | Hypothetical protein | 2.08 |
| AFUA_5G12220 |  | Hypothetical protein | 1.91 |
| AFUA_1G13810 |  | Hypothetical protein | 1.83 |
| AFUA_6G14410 |  | Amidase family protein | 1.76 |
| AFUA_6G07040 | *atg5* | Putative autophagy protein | 1.75 |
| AFUA_1G17340 |  | Putative dioxygenase | 1.73 |
| AFUA_4G00740 |  | Hypothetical protein | 1.66 |
| AFUA_2G00910 |  | Pfs, NB-ARC and TPR domain protein | 1.65 |
| AFUA_3G02260 |  | Hypothetical protein | 1.64 |
| AFUA_7G06720 |  | Putative tannase | 1.63 |
| AFUA_2G16462 | *Tfc3* | Putative TFIIIC transcription initiation factor complex subunits | 1.63 |
| AFUA_3G13190 |  | Hypothetical protein | 1.63 |
| AFUA_8G01590 |  | Hypothetical protein | 1.61 |
| AFUA_2G01860 |  | Integral membrane protein | 1.56 |
| AFUA_3G00630 |  | Hypothetical protein | 1.54 |
| AFUA_6G00600 |  | Hypothetical protein | 1.54 |
| AFUA_6G07050 |  | Putative Na+/H+ antiporter | 1.53 |
| AFUA_2G09460 |  | Potassium transporter | 1.53 |
| AFUA_7G06750 |  | Putative phosphoglycerate mutase family protein | 1.49 |
| AFUA_6G02845 | *EutQ* | Putative ethanolamine utilization protein | 1.45 |
| AFUA_4G06980 |  | Hypothetical protein | 1.45 |
| AFUA_3G12280 | *Translin* | Putative recombination hotspot-binding protein | 1.43 |
| AFUA_4G03190 | *tpsC* | Putative alpha, alpha-trehalose-phosphate synthase subunit | 1.43 |
| AFUA_5G09280 |  | DUF1479 domain protein | 1.41 |
| AFUA_3G02890 |  | Putative MFS sugar transporter | 1.40 |
| AFUA_7G06730 |  | Putative FAD monooxygenase | 1.35 |
| AFUA_4G04320 |  | Putative homeobox transcription factor | 1.34 |
| AFUA_3G00170 |  | Hypothetical protein | 1.31 |
| AFUA_1G05360 |  | CAIB/BAIF family enzyme | 1.31 |
| AFUA_6G02810 |  | Putative low-affinity copper transporter | 1.29 |
| AFUA_6G13640 |  | Hypothetical protein | 1.29 |
| AFUA_2G15720 |  | Hypothetical protein | 1.24 |
| AFUA_1G01520 |  | Hypothetical protein | 1.22 |
| AFUA_2G17210 |  | Hypothetical protein | 1.22 |
| AFUA_1G00670 |  | Hypothetical protein | 1.17 |
| AFUA_7G08320 |  | Putative heat shock transcription factor | 1.17 |
| AFUA_3G02800 |  | Putative lipase/esterase | 1.17 |
| AFUA_1G07000 | *Rad26* | Putative DNA repair protein | 1.13 |
| AFUA_4G04605 |  | Hypothetical protein | 1.11 |
| AFUA_2G00550 |  | Hypothetical protein | 1.08 |
| AFUA_1G11230 | *hmg2* | HMG-CoA reductase | 1.08 |
| AFUA_3G13560 |  | Putative toxin biosynthesis protein | 1.03 |
| AFUA_3G03270 |  | Putative isochorismatase family hydrolase | 1.01 |

**Table S2. Core 35 up-regulated DEGs in *A. fumigatus* on exposure to NP**

| Gene ID | Gene Name | Description | log2 Fold Change |
| --- | --- | --- | --- |
| AFUA_6G11770 |  | NmrA-like family protein | 3.62 |
| AFUA_3G00620 |  | Putative zinc-containing alcohol dehydrogenase | 3.33 |
| AFUA_3G03080 | *eng7* | Putative endo-1,3(4)-beta-glucanase | 2.86 |
| AFUA_3G02710 |  | Putative zinc-binding oxidoreductase | 2.72 |
| AFUA_2G17720 |  | PAF acetylhydrolase family protein | 2.38 |
| AFUA_3G02790 |  | amidohydrolase family protein | 1.95 |
| AFUA_7G06180 |  | Hypothetical protein | 1.90 |
| AFUA_8G00540 | *nrps14* | Putative hybrid PKS-NRPS enzyme | 1.85 |
| AFUA_5G09900 |  | Putative RTA1 domain protein | 1.81 |
| AFUA_2G00700 |  | Hypothetical protein | 1.77 |
| AFUA_8G06540 |  | Putative ornithine decarboxylase | 1.72 |
| AFUA_8G06320 |  | Hypothetical protein | 1.72 |
| AFUA_1G13830 |  | Threonine-rich protein | 1.67 |
| AFUA_8G06040 |  | Hypothetical protein | 1.64 |
| AFUA_3G09230 |  | Putative carboxylesterase | 1.59 |
| AFUA_2G17600 | *pksP* | Conidial pigment polyketide synthase PksP/Alb1 | 1.49 |
| AFUA_7G01130 |  | Hypothetical protein | 1.48 |
| AFUA_3G07630 |  | Metallo-beta-lactamase superfamily protein | 1.46 |
| AFUA_5G09800 |  | Putative hydrolase | 1.43 |
| AFUA_2G15920 |  | Hypothetical protein | 1.33 |
| AFUA_8G06405 |  | Isoflavone reductase family protein | 1.30 |
| AFUA_4G14540 | *tpcE* | Trypacidin cluster transcription factor | 1.25 |
| AFUA_3G13020 |  | Hypothetical protein | 1.18 |
| AFUA_3G09120 |  | Hypothetical protein | 1.15 |
| AFUA_5G14610 | *cp8* | Putative carboxypeptidase Y | 1.11 |
| AFUA_3G01600 |  | Putative salicylate hydroxylase | 1.07 |
| AFUA_1G16140 |  | Hypothetical protein | 1.05 |
| AFUA_3G00460 |  | Hypothetical protein | 1.05 |
| AFUA_5G00830 |  | Hypothetical protein | 1.05 |
| AFUA_3G13550 |  | Putative 2-dehydropantoate 2-reductase family protein | 1.04 |
| AFUA_6G02570 |  | Hypothetical protein | 1.02 |
| AFUA_2G00710 |  | Putative alpha-amylase | 1.02 |
| AFUA_1G14945 |  | Putative C6 transcription factor | 1.01 |
| AFUA_2G15990 |  | Hypothetical protein | 1.00 |
| AFUA_2G00650 |  | Hypothetical protein | 1.00 |

**Table S3. Core 1 down-regulated DEG in *A. fumigatus* on exposure to NP**

| Gene ID | Gene Name | Description | log2 Fold Change |
| --- | --- | --- | --- |
| AFUA_1G03210 | *flbD* | MYB family conidiophore development protein | -2.27 |

**Table S4. Core 327 down-regulated DEGs in *A. fumigatus* on exposure to NP**

| Gene ID | Gene Name | Description | log2 Fold Change |
| --- | --- | --- | --- |
| AFUA_6G03690 | *ena1* | Putative P-type ATPase sodium pump | -9.06 |
| AFUA_4G01560 |  | Putative MFS myo-inositol transporter | -7.88 |
| AFUA_4G09440 |  | Putative P-type ATPase sodium transporter | -5.85 |
| AFUA_4G03710 |  | Putative glutamine synthetase | -5.68 |
| AFUA_2G00210 |  | Hypothetical protein | -5.35 |
| AFUA_2G11830 |  | Hypothetical protein | -5.31 |
| AFUA_7G00580 |  | Putative adhesin | -5.30 |
| AFUA_4G03700 |  | GMP synthase | -5.16 |
| AFUA_4G09840 | *bna5-2* | Putative kynureninase 2 | -5.06 |
| AFUA_8G01030 |  | Hypothetical protein | -4.91 |
| AFUA_3G01370 |  | Putative MFS transporter | -4.76 |
| AFUA_4G03690 |  | Putative aldehyde dehydrogenase family protein | -4.73 |
| AFUA_2G12530 |  | Putative carnitine acetyl transferase | -4.48 |
| AFUA_7G01010 | *adh1* | Putative alcohol dehydrogenase | -4.48 |
| AFUA_8G02550 |  | Putative MFS peptide transporter | -4.40 |
| AFUA_7G01000 |  | Putative alcohol dehydrogenase | -4.31 |
| AFUA_8G02560 |  | Putative glyceraldehyde-3-phosphate dehydrogenase | -4.21 |
| AFUA_4G03900 | *mfp* | Putative multifunctional beta-oxidation protein | -4.11 |
| AFUA_8G07080 | *mep* | Putative secreted metalloprotease | -4.08 |
| AFUA_4G03680 |  | Putative oxidoreductase, short-chain dehydrogenase/reductase family | -4.00 |
| AFUA_8G05710 | *mfsA* | Putative MFS sugar transporter | -3.94 |
| AFUA_8G01010 |  | Thermophilic desulfurizing enzyme family protein | -3.79 |
| AFUA_2G04230 | *fahA* | Putative fumarylacetoacetase | -3.64 |
| AFUA_4G09830 |  | Indoleamine 2,3-dioxygenase pyrrole 2,3-dioxygenase, role in 'de novo' NAD biosynthetic process from tryptophan, tryptophan catabolic process to kynurenine and cytoplasm localization | -3.61 |
| AFUA_4G00800 |  | Putative monosaccharide transporter | -3.55 |
| AFUA_8G01020 |  | HET domain protein | -3.41 |
| AFUA_4G12020 |  | Hypothetical protein | -3.37 |
| AFUA_4G09320 | *dppIV* | Putative extracellular dipeptidyl-peptidase | -3.37 |
| AFUA_3G03030 |  | Putative catechol dioxygenase | -3.32 |
| AFUA_6G02210 |  | Putative cytochrome P450 monooxygenase | -3.31 |
| AFUA_8G05530 | *osm1* | Putative fumarate reductase | -3.31 |
| AFUA_2G01320 |  | Putative potassium/sodium P-type ATPase | -3.27 |
| AFUA_4G12870 |  | Putative methylmalonate-semialdehyde dehydrogenase | -3.26 |
| AFUA_6G02215 |  | Hypothetical protein | -3.25 |
| AFUA_1G02770 |  | Hypothetical protein | -3.25 |
| AFUA_5G08940 | *MccB* | 3-methylcrotonyl-CoA carboxylase, beta subunit | -3.19 |
| AFUA_7G06090 | *pox1* | Putative fatty-acyl coenzyme A oxidase | -3.18 |
| AFUA_4G10950 |  | 3-ketoacyl-coA thiolase peroxisomal A precursor | -3.18 |
| AFUA_3G03060 | *aspf34* | cell wall protein PhiA, Allergen | -3.17 |
| AFUA_5G08910 | *mccA* | Putative 3-methylcrotonyl-CoA carboxylase subunit alpha | -3.13 |
| AFUA_8G02315 |  | Hypothetical protein | -3.10 |
| AFUA_7G06100 |  | Acyl-CoA dehydrogenase family protein | -3.06 |
| AFUA_3G11790 |  | Putative galactose-proton symport | -3.06 |
| AFUA_2G10230 |  | Putative inositol oxygenase | -3.01 |
| AFUA_6G03700 |  | Hypothetical protein | -3.00 |
| AFUA_8G06870 |  | Putative MFS sugar transporter | -2.99 |
| AFUA_8G01890 |  | Putative Na+/H+ exchanger family protein | -2.98 |
| AFUA_8G02010 |  | Putative MFS sugar transporter | -2.95 |
| AFUA_2G04220 | *hmgA* | Homogentisate 1,2-dioxygenase | -2.94 |
| AFUA_4G12010 |  | Putative 2-oxo acid dehydrogenases acyltransferase | -2.92 |
| AFUA_3G01450 |  | Putative 3-methyl-2-oxobutanoate dehydrogenase | -2.92 |
| AFUA_6G10880 |  | Putative acyl-CoA dehydrogenase family protein | -2.91 |
| AFUA_7G01690 |  | Putative acetamidase | -2.82 |
| AFUA_4G08710 |  | Putative short chain dehydrogenase | -2.81 |
| AFUA_4G12030 |  | Hypothetical protein | -2.78 |
| AFUA_2G10220 |  | Putative glycerol dehydrogenase | -2.78 |
| AFUA_1G14850 | *cybS* | Putative acyl-CoA dehydrogenase | -2.75 |
| AFUA_1G16690 |  | Putative MFS sugar transporter | -2.75 |
| AFUA_6G14330 |  | Putative 5-oxo-L-prolinase | -2.73 |
| AFUA_7G01090 | *PrnB* | Putative proline permease | -2.70 |
| AFUA_5G06680 | *gatA* | 4-aminobutyrate aminotransferase | -2.69 |
| AFUA_4G12880 |  | Hypothetical protein | -2.68 |
| AFUA_3G03450 |  | Putative cytochrome P450 oxidoreductase | -2.67 |
| AFUA_6G03590 | *mcsA* | Methylcitrate synthase | -2.65 |
| AFUA_2G10160 |  | Putative phenylacetyl-CoA ligase | -2.62 |
| AFUA_6G13440 |  | Putative choline sulfatase | -2.61 |
| AFUA_7G01400 | *BimC* | Putative kinesin family protein | -2.61 |
| AFUA_6G14100 |  | Putative mitochondrial carnitine:acyl carnitine carrier | -2.58 |
| AFUA_6G00430 |  | IgE-binding protein | -2.55 |
| AFUA_1G15170 |  | acyl-CoA thioesterase II | -2.53 |
| AFUA_2G01310 |  | Putative EF-hand calcium-binding domain protein | -2.51 |
| AFUA_1G12300 |  | Putative acyl CoA binding protein | -2.50 |
| AFUA_4G01510 |  | Putative C6 transcription factor | -2.49 |
| AFUA_6G09310 |  | Putative class V chitinase | -2.48 |
| AFUA_7G06370 |  | Putative C6 transcription factor | -2.46 |
| AFUA_1G01590 |  | Putative C6 transcription factor | -2.42 |
| AFUA_1G12310 |  | Putative GABA permease | -2.40 |
| AFUA_4G02870 |  | Putative 2-ketogluconate transporter | -2.40 |
| AFUA_1G04780 | *pxa1* | Putative peroxisomal ABC transporter | -2.38 |
| AFUA_6G09305 |  | Hypothetical protein | -2.37 |
| AFUA_5G08930 | *IvdA* | Putative isovaleryl-CoA dehydrogenase | -2.37 |
| AFUA_2G04200 | *hppD* | 4-hydroxyphenylpyruvate dioxygenase | -2.36 |
| AFUA_6G14200 | *erg10* | Putative acetyl-CoA acetyltransferase | -2.35 |
| AFUA_1G14330 | *abcC* | Putative ABC transporter, Azole transporter | -2.35 |
| AFUA_8G02000 |  | Putative sorbitol/xylitol dehydrogenase | -2.33 |
| AFUA_5G04270 |  | Putative AMP-binding enzyme | -2.32 |
| AFUA_5G14210 | *grg1* | Glucose repressible protein | -2.32 |
| AFUA_6G12250 | *ScoT* | Putative succinyl-CoA:3-ketoacid-coenzyme A transferase | -2.29 |
| AFUA_5G08470 |  | Putative AMP-binding enzyme | -2.27 |
| AFUA_6G00750 | *pdcB* | Putative pyruvate decarboxylase | -2.26 |
| AFUA_5G10280 |  | Oxidoreductase, acting on the CH-OH group of donors, NAD or NADP as acceptor | -2.26 |
| AFUA_7G00950 |  | Putative MFS monosaccharide transporter | -2.25 |
| AFUA_2G12500 |  | Putative MFS multidrug transporter | -2.25 |
| AFUA_3G01840 |  | Putative MFS transporter | -2.25 |
| AFUA_2G13770 | *flbC* | Putative C2H2 zinc finger transcription factor | -2.25 |
| AFUA_8G05240 | *pex5* | Putative peroxisomal targeting signal receptor | -2.24 |
| AFUA_5G00640 |  | Putative peroxisomal dehydratase | -2.24 |
| AFUA_2G04240 | *maiA* | Putative maleylacetoacetate isomerase | -2.23 |
| AFUA_6G11430 | *aldA* | Putative aldehyde dehydrogenase | -2.20 |
| AFUA_7G06080 |  | Putative ubiE/COQ5 methyltransferase | -2.19 |
| AFUA_5G08920 |  | Hypothetical protein | -2.19 |
| AFUA_5G00290 |  | Putative C6 transcription factor | -2.17 |
| AFUA_7G04260 | *pex10* | Putative peroxisome biosynthesis protein | -2.16 |
| AFUA_6G00120 |  | Putative C6 transcription factor | -2.16 |
| AFUA_8G04130 | *farB1* | Fungal Zn(2)-Cys(6) binuclear cluster domain-containing protein | -2.16 |
| AFUA_2G04210 | *hmgX* | L-tyrosine degradation gene cluster protein | -2.15 |
| AFUA_6G03060 |  | Putative MFS monosaccharide transporter | -2.14 |
| AFUA_4G11700 |  | Leucine Rich Repeat domain protein | -2.13 |
| AFUA_4G13380 |  | Hypothetical protein | -2.13 |
| AFUA_8G07230 |  | Putative C6 finger domain protein | -2.11 |
| AFUA_6G11930 |  | Hypothetical protein | -2.11 |
| AFUA_8G05700 |  | Hypothetical protein | -2.11 |
| AFUA_8G02200 |  | Putative proline permease | -2.10 |
| AFUA_1G12650 | *kat1* | Putative 3-ketoacyl-CoA ketothiolase | -2.09 |
| AFUA_8G07360 |  | Putative C6 transcription factor | -2.09 |
| AFUA_2G10170 |  | Hypothetical protein | -2.09 |
| AFUA_4G01350 | *gprK* | G-protein coupled receptor | -2.09 |
| AFUA_2G00180 |  | Neutral amino acid permease | -2.09 |
| AFUA_2G04080 |  | GPR/FUN34 family protein | -2.06 |
| AFUA_8G02250 |  | Hypothetical protein | -2.06 |
| AFUA_6G00760 |  | Predicted glutathione S transferase | -2.05 |
| AFUA_4G03750 |  | Putative phthalate transporter | -2.05 |
| AFUA_6G13280 |  | Putative lipid transfer protein | -2.04 |
| AFUA_5G06410 | *AmdA* | Putative C2H2 transcription factor | -2.04 |
| AFUA_6G13570 |  | Putative cytochrome c peroxidase | -2.03 |
| AFUA_4G08490 |  | Putative acyl-CoA dehydrogenase | -2.03 |
| AFUA_4G01220 |  | Hypothetical protein | -2.02 |
| AFUA_6G03730 |  | 2-methylcitrate dehydratase | -2.02 |
| AFUA_6G03720 |  | Putative MFS allantoate transporter | -2.02 |
| AFUA_2G09850 |  | Putative oxidoreductase, 2-nitropropane dioxygenase family | -2.02 |
| AFUA_6G08700 | *exg20* | Putative beta glucosidase | -2.01 |
| AFUA_4G03670 |  | Putative C6 transcription factor | -2.01 |
| AFUA_4G00150 |  | Putative MFS maltose transporter | -2.01 |
| AFUA_3G11970 | *pacC* | C2H2 finger domain transcription factor | -1.99 |
| AFUA_2G04262 |  | Putative C6 transcription factor | -1.99 |
| AFUA_7G01100 |  | Hypothetical protein | -1.97 |
| AFUA_2G16260 | *Ase1* | Putative microtubule associated protein | -1.96 |
| AFUA_4G03960 | *farA* | Fungal Zn(2)-Cys(6) binuclear cluster domain-containing protein | -1.96 |
| AFUA_3G09640 |  | Putative cAMP independent regulatory protein | -1.94 |
| AFUA_5G14060 | *rho4* | Putative Rho-type GTPase | -1.94 |
| AFUA_5G10290 |  | Putative fructose-bisphosphate aldolase | -1.93 |
| AFUA_3G10110 |  | Electron transfer flavoprotein-ubiquinone oxidoreductase | -1.93 |
| AFUA_6G07740 | *pex11* | Putative peroxisome biogenesis factor | -1.92 |
| AFUA_4G04180 | *chsB* | Putative class II chitin synthase | -1.91 |
| AFUA_6G02860 | *acuD* | Putative isocitrate lyase | -1.90 |
| AFUA_4G06100 | *pex20* | Putative peroxisome biogenesis protein | -1.90 |
| AFUA_6G13830 |  | Oxidoreductase, short chain dehydrogenase/reductase family | -1.88 |
| AFUA_3G11650 |  | Hypothetical protein | -1.88 |
| AFUA_5G02840 |  | Putative MFS sugar transporter | -1.88 |
| AFUA_6G14190 |  | Hypothetical protein | -1.88 |
| AFUA_8G02450 |  | Hypothetical protein | -1.88 |
| AFUA_2G10205 |  | Hypothetical protein | -1.87 |
| AFUA_6G08750 | *prnC* | Putative delta-1-pyrroline-5-carboxylate dehydrogenase | -1.87 |
| AFUA_6G08830 |  | 2-oxoisovalerate dehydrogenase complex alpha subunit | -1.86 |
| AFUA_2G11900 |  | Putative pyruvate dehydrogenase kinase | -1.86 |
| AFUA_4G09510 |  | Hypothetical protein | -1.86 |
| AFUA_4G05900 |  | Hypothetical protein | -1.84 |
| AFUA_1G03560 | *pex2* | Putative peroxisomal biogenesis factor 2 | -1.84 |
| AFUA_6G14180 | *Alg1* | Putative beta-1,4-mannosyltransferase | -1.83 |
| AFUA_6G00680 |  | Hypothetical protein | -1.82 |
| AFUA_4G10970 |  | Hypothetical protein | -1.82 |
| AFUA_5G08900 | *ArbD* | Putative d-arabitinol dehydrogenase | -1.82 |
| AFUA_2G16730 | *pex12* | Peroxisome biosynthesis protein (PAS10/Peroxin-12) | -1.81 |
| AFUA_2G00790 |  | Hypothetical protein | -1.81 |
| AFUA_2G08470 | *bud4* | GTP binding protein | -1.81 |
| AFUA_8G05680 |  | Putative serine/threonine protein kinase | -1.80 |
| AFUA_8G04780 | *PmpP24* | Putative peroxisomal membrane protein | -1.79 |
| AFUA_6G09320 |  | Putative LysM domain protein | -1.79 |
| AFUA_6G14110 |  | Hypothetical protein | -1.79 |
| AFUA_7G05580 | *pldA* | Putative phospholipase D | -1.78 |
| AFUA_2G11890 |  | Hypothetical protein | -1.78 |
| AFUA_1G16710 | *Gig30* | Putative fatty acid elongase | -1.77 |
| AFUA_4G04410 |  | Putative 3-hydroxybutyryl-CoA dehydrogenase, Immunoreactive protein | -1.76 |
| AFUA_8G05160 | *pex13* | Putative peroxisomal membrane protein 13 | -1.76 |
| AFUA_3G10190 | *pex14* | Putative peroxisomal membrane protein 14 | -1.76 |
| AFUA_4G10940 | *DlpA* | Dynamin-like protein A domain protein | -1.74 |
| AFUA_8G02210 |  | Alpha-ketoglutarate-dependent taurine dioxygenase | -1.74 |
| AFUA_3G14520 |  | Enoyl-CoA hydratase/isomerase family protein | -1.74 |
| AFUA_7G06390 | *MalP* | Putative MFS alpha-glucoside transporter, MFS maltose permease | -1.74 |
| AFUA_4G13510 | *acuD* | Isocitrate lyase | -1.73 |
| AFUA_7G00780 |  | Putative MFS monocarboxylate transporter | -1.73 |
| AFUA_4G03730 |  | Dienelactone hydrolase | -1.71 |
| AFUA_1G10220 |  | Hypothetical protein | -1.71 |
| AFUA_2G10920 | *echA* | Enoyl-CoA hydratase/isomerase family protein | -1.71 |
| AFUA_4G12780 |  | Hypothetical protein | -1.70 |
| AFUA_6G09315 |  | Hypothetical protein | -1.69 |
| AFUA_1G04160 |  | Putative aspartate aminotransferase | -1.66 |
| AFUA_4G12040 | *erg7B* | Putative lanosterol synthase | -1.66 |
| AFUA_2G08410 | *pex8* | Putative peroxisomal biogenesis factor 8 | -1.66 |
| AFUA_2G02080 |  | Putative C2H2 finger domain protein | -1.65 |
| AFUA_1G06330 |  | Hypothetical protein | -1.65 |
| AFUA_1G07610 | *pex16* | Putative peroxisomal membrane protein | -1.64 |
| AFUA_6G03600 | *Pth11* | Putative integral membrane protein | -1.64 |
| AFUA_3G12820 |  | Kinesin family protein | -1.62 |
| AFUA_2G12600 |  | Hypothetical protein | -1.61 |
| AFUA_1G01580 |  | Hypothetical protein | -1.61 |
| AFUA_6G03540 | *acuE* | Putative malate synthase | -1.61 |
| AFUA_3G10250 | *Cdc15* | Putative cell division control protein | -1.61 |
| AFUA_4G11800 | *alp1* | Putative secreted alkaline serine protease | -1.61 |
| AFUA_1G03150 | *erg24* | Putative C-14 sterol reductase | -1.61 |
| AFUA_6G13330 |  | Putative RNA binding protein of unknown function | -1.60 |
| AFUA_3G01860 |  | Hypothetical protein | -1.59 |
| AFUA_8G02190 |  | Hypothetical protein | -1.59 |
| AFUA_3G03445 |  | Putative short-chain alcohol dehydrogenase | -1.59 |
| AFUA_2G01610 |  | Hypothetical protein | -1.59 |
| AFUA_6G00130 |  | Putative MFS transporter | -1.57 |
| AFUA_1G00510 | *dld1* | D-lactate dehydrogenase (cytochrome) | -1.56 |
| AFUA_5G02780 |  | Putative mitochondrial nicotinamide nucleotide transhydrogenase subunit | -1.56 |
| AFUA_2G11880 |  | Hypothetical protein | -1.56 |
| AFUA_5G06670 |  | Integral membrane protein | -1.55 |
| AFUA_4G12050 |  | Thermoresistant gluconokinase | -1.55 |
| AFUA_4G11110 |  | C2 domain protein | -1.55 |
| AFUA_4G09140 | *car2* | L-ornithine aminotransferase | -1.54 |
| AFUA_3G03852 |  | C2H2 type zinc finger domain protein | -1.53 |
| AFUA_2G01600 |  | Putative sphingomyelin phosphodiesterase | -1.53 |
| AFUA_4G14450 | *M2DH* | Mannitol 2-dehydrogenase | -1.53 |
| AFUA_4G03722 | *DSD1* | D-serine ammonia-lyase | -1.52 |
| AFUA_2G10240 |  | Putative NAD binding Rossmann fold oxidoreductase | -1.52 |
| AFUA_7G02570 | *TinC* | NIMA-interacting protein | -1.52 |
| AFUA_6G13840 |  | Hypothetical protein | -1.51 |
| AFUA_4G00860 | *dprA* | Dehydrin-like protein, cell surface protein | -1.51 |
| AFUA_1G10780 |  | Glycine cleavage system T protein | -1.49 |
| AFUA_2G04140 | *Spc105* | Putative chromosome segregation protein | -1.48 |
| AFUA_8G00720 |  | Putative amino acid transporter | -1.48 |
| AFUA_3G11920 |  | Dihydrodipicolinate synthetase family protein | -1.48 |
| AFUA_6G04040 |  | Peroxisomal D3,D2-enoyl-CoA isomerase | -1.47 |
| AFUA_2G05450 |  | Putative NADH-ubiquinone oxidoreductase 64 kDa subunit | -1.45 |
| AFUA_5G07470 |  | Oxidoreductase, short-chain dehydrogenase/reductase family | -1.45 |
| AFUA_3G12250 | *cdcA* | Putative tyrosine-protein phosphatase CDC14 | -1.45 |
| AFUA_3G10180 |  | HEC/Ndc80p family protein | -1.45 |
| AFUA_5G12580 |  | Putative GTP binding protein (GTPBP1) | -1.44 |
| AFUA_2G12550 |  | Putative MFS multidrug transporter | -1.43 |
| AFUA_1G03440 |  | Putative peroxisomal carrier protein | -1.43 |
| AFUA_7G01102 |  | Hypothetical protein | -1.42 |
| AFUA_3G09990 |  | Hypothetical protein | -1.42 |
| AFUA_4G03240 | *Mp1* | Cell wall serine-threonine-rich galactomannoprotein | -1.41 |
| AFUA_1G05870 | *Scs3* | Putative inositol phospholipid biosynthesis protein | -1.40 |
| AFUA_3G13830 |  | PH domain protein | -1.40 |
| AFUA_1G01370 |  | Predicted glutathione S transferase | -1.39 |
| AFUA_4G09150 |  | Putative ABC multidrug transporter | -1.38 |
| AFUA_4G08730 |  | Hypothetical protein | -1.38 |
| AFUA_5G09450 |  | Isoflavone reductase family protein | -1.36 |
| AFUA_5G06120 | *Rfx1* | Putative DNA damage and replication checkpoint protein | -1.36 |
| AFUA_1G16810 |  | Aminotransferase, class III | -1.35 |
| AFUA_2G03640 |  | Ras GTPase activating protein | -1.35 |
| AFUA_1G17370 | *scf1* | Putative heat shock protein | -1.35 |
| AFUA_3G00440 |  | F-box domain protein | -1.34 |
| AFUA_1G02570 | *erdS* | Ergosteryl-3beta-O-aspartate synthase | -1.34 |
| AFUA_5G11980 |  | MFS efflux transporter | -1.33 |
| AFUA_7G04200 |  | Hypothetical protein | -1.33 |
| AFUA_6G14370 |  | Putative AraC-like ligand binding domain protein | -1.33 |
| AFUA_2G00340 |  | Putative diaminopropionate ammonia-lyase | -1.33 |
| AFUA_7G05730 |  | Hypothetical protein | -1.33 |
| AFUA_1G14880 |  | N-acylethanolamine amidohydrolase | -1.31 |
| AFUA_4G09970 |  | Hypothetical protein | -1.30 |
| AFUA_8G04140 |  | Hypothetical protein | -1.29 |
| AFUA_6G12180 | *dprB* | Dehydrin-like protein | -1.28 |
| AFUA_2G00360 |  | Putative homeobox and C2H2 transcription factor | -1.28 |
| AFUA_6G09530 |  | Hypothetical protein | -1.28 |
| AFUA_3G14260 |  | Putative mismatched base pair and cruciform DNA recognition protein | -1.27 |
| AFUA_6G00440 |  | Putative cation diffusion facilitator | -1.27 |
| AFUA_1G17350 |  | Putative CP2 transcription factor | -1.27 |
| AFUA_3G08880 |  | Hypothetical protein | -1.27 |
| AFUA_8G05040 |  | Dihydrodipicolinate synthetase family protein | -1.27 |
| AFUA_2G10150 | *pex1* | Putative peroxisome biosynthesis protein | -1.26 |
| AFUA_5G04336 |  | Hypothetical protein | -1.26 |
| AFUA_4G04190 |  | Hypothetical protein | -1.26 |
| AFUA_3G03910 |  | IQ calmodulin-binding motif protein | -1.26 |
| AFUA_5G13800 |  | Putative transcriptional regulator | -1.26 |
| AFUA_2G17610 |  | Sulfatase domain protein | -1.26 |
| AFUA_4G03120 |  | Putative mitochondrial cytochrome b2 | -1.26 |
| AFUA_6G02670 | *nimA* | Putative cell-cycle regulated serine/threonine protein kinase, G2-specific protein kinase | -1.24 |
| AFUA_5G02480 | *Gsy1* | Putative glycogen synthase | -1.24 |
| AFUA_1G14730 | *Cdc20* | Putative cell division cycle protein | -1.24 |
| AFUA_8G01990 |  | Putative C6 transcription factor | -1.24 |
| AFUA_4G11730 | *gldB* | Putative glycerol dehydrogenase | -1.24 |
| AFUA_4G03000 | *nte1* | Putative lysophospholipase, patatin-like serine hydrolase | -1.24 |
| AFUA_5G02220 |  | Putative mammalian Ste20-like protein kinase 3 (Mst3)-like protein kinase | -1.23 |
| AFUA_4G08440 |  | Patatin-like serine hydrolase | -1.23 |
| AFUA_4G02790 |  | Putative carbon-nitrogen family hydrolase | -1.23 |
| AFUA_5G13100 |  | Hypothetical protein | -1.23 |
| AFUA_8G05010 | *zfpA* | Putative C2H2 zinc-finger transcription factor | -1.23 |
| AFUA_8G04920 |  | Late embryogenesis abundant (LEA) domain protein | -1.22 |
| AFUA_3G07990 | *GabA* | GABA permease | -1.22 |
| AFUA_6G12980 |  | Putative spindle pole body component | -1.22 |
| AFUA_2G05310 |  | Putative C6 transcription factor | -1.22 |
| AFUA_3G08000 | *pex6* | Putative peroxisomal ATPase | -1.21 |
| AFUA_1G15380 |  | Hypothetical protein | -1.21 |
| AFUA_8G05580 | *coaT* | Putative coenzyme A transferas | -1.21 |
| AFUA_1G14390 |  | Putative zinc-dependent alcohol dehydrogenase | -1.20 |
| AFUA_7G06770 |  | Hypothetical protein | -1.20 |
| AFUA_3G08750 |  | Hypothetical protein | -1.18 |
| AFUA_4G11720 |  | Putative phosphatidyl synthase | -1.18 |
| AFUA_2G11990 |  | Putative phosphoinositide phosphatase Pten/Tep1 with a predicted role in lipid metabolism | -1.18 |
| AFUA_3G08870 |  | Putative phytase, phoB-regulated | -1.17 |
| AFUA_2G14590 |  | Putative MFS monosaccharide transporter | -1.16 |
| AFUA_6G10260 | *akr1* | Putative aldehyde reductase | -1.16 |
| AFUA_6G08200 | *nimT*  *Mih1* | Putative M-phase inducer phosphatase  Putative cell cycle control protein tyrosine phosphatase | -1.15 |
| AFUA_4G12590 |  | Conidia-enriched protein of unknown function | -1.15 |
| AFUA_4G04390 | *tof1* | Putative topoisomerase 1-associated factor 1, DNA repair protein | -1.14 |
| AFUA_2G13270 |  | Putative alcohol dehydrogenase | -1.14 |
| AFUA_8G02480 |  | Hypothetical protein | -1.13 |
| AFUA_6G08530 | *Src1* | Putative sister chromatid separation protein | -1.12 |
| AFUA_1G00500 |  | Putative FMN dependent dehydrogenase | -1.12 |
| AFUA_4G02970 | *Aqy1*  *TMEM16* | Putative plasma membrane channel protein, Dual function calcium channel/ scramblase protein | -1.12 |
| AFUA_5G02490 |  | Putative zinc-binding oxidoreductase | -1.11 |
| AFUA_6G09520 |  | Lipase/esterase family protein | -1.11 |
| AFUA_2G04540 |  | Hypothetical protein | -1.11 |
| AFUA_3G13140 |  | Putative methyltransferase | -1.11 |
| AFUA_8G01970 | *pgaB* | Putative extracellular endo-polygalacturonase | -1.10 |
| AFUA_3G00130 |  | Hypothetical protein | -1.10 |
| AFUA_8G06760 |  | Putative MFS transporter | -1.09 |
| AFUA_1G16580 |  | Hypothetical protein | -1.09 |
| AFUA_3G11250 | *ace2* | Conidiophore development regulator | -1.08 |
| AFUA_7G04520 | *dprC* | Dehydrin-like protein | -1.08 |
| AFUA_2G02170 | *smc4* | Putative nuclear condensin complex subunit | -1.07 |
| AFUA_1G01850 |  | Putative C6 finger domain protein | -1.07 |
| AFUA_2G07550 | *Ark1* | Putative serine/threonine protein kinase | -1.07 |
| AFUA_4G08170 | *uga2* | Putative succinate-semialdehyde dehydrogenase | -1.07 |
| AFUA_6G08120 | *SldA* | Putative checkpoint protein kinase | -1.07 |
| AFUA_3G06660 |  | NIPSNAP family protein | -1.07 |
| AFUA_2G00350 | *ArgE* | Putative acetylornithine deacetylase | -1.06 |
| AFUA_3G12330 |  | Putative phosphatidyl synthase | -1.06 |
| AFUA_1G04300 |  | Hypothetical protein | -1.06 |
| AFUA_2G03150 |  | Kinesin family protein | -1.06 |
| AFUA_6G03010 | *Zms1* | Putative C2H2 finger domain protein | -1.03 |
| AFUA_4G11310 | *fbp1* | Fructose-1,6-bisphosphatase | -1.01 |

**Table S5. Core 189 down-regulated DEGs in *A. fumigatus* on exposure to NP**

| Gene ID | Gene Name | Description | log2 Fold Change |
| --- | --- | --- | --- |
| AFUA_7G00990 | *AlcS* | Transcriptional activator of ethanol catabolism | -5.51 |
| AFUA_6G14560 |  | Putative hexose transporter | -5.16 |
| AFUA_6G11910 | *exg19* | Putative beta-glucosidase | -4.65 |
| AFUA_5G00310 |  | Putative flavin-containing monooxygenase | -4.00 |
| AFUA_4G09420 | *cafC* | Putative carbonic anhydrase | -3.11 |
| AFUA_6G09680 | *gliM* | O-methyltransferase | -2.89 |
| AFUA_4G00750 |  | Hypothetical protein | -2.58 |
| AFUA_8G00980 |  | Hypothetical protein | -2.49 |
| AFUA_7G00570 |  | Hypothetical protein | -2.45 |
| AFUA_2G14540 |  | Putative endoglucanase | -2.36 |
| AFUA_1G01960 |  | Hypothetical protein | -2.35 |
| AFUA_2G08810 |  | Hypothetical protein | -2.28 |
| AFUA_4G01570 |  | Putative amino acid transporter | -2.25 |
| AFUA_2G00500 |  | Hypothetical protein | -2.24 |
| AFUA_6G00140 |  | Hypothetical protein | -2.23 |
| AFUA_2G08800 | *Dip5* | Putative amino acid permease | -2.13 |
| AFUA_3G00810 | *hyd1* | Putative cholestenol delta-isomerase | -2.05 |
| AFUA_7G06920 |  | Putative NmrA family transcriptional regulator | -1.93 |
| AFUA_6G00770 |  | Putative extracellular arabinanase | -1.91 |
| AFUA_6G11390 | *gel2* | GPI-anchored 1,3-beta-glucanosyltransferase | -1.89 |
| AFUA_5G02820 |  | Hypothetical protein | -1.82 |
| AFUA_3G01872 |  | Hypothetical protein | -1.80 |
| AFUA_7G00280 |  | Hypothetical protein | -1.80 |
| AFUA_3G01400 |  | Putative ABC multidrug transporter | -1.79 |
| AFUA_1G14860 |  | Putative bZIP transcription factor | -1.70 |
| AFUA_5G10210 |  | Hypothetical protein | -1.69 |
| AFUA_7G00652 |  | Putative C6 and C2H2 transcription factor RegA-like | -1.64 |
| AFUA_4G03200 |  | Hypothetical protein | -1.63 |
| AFUA_3G06730 |  | Putative MFS sugar transporter | -1.63 |
| AFUA_3G01670 |  | Putative MFS hexose transporter | -1.61 |
| AFUA_8G01850 |  | Putative phosphate-repressible phosphate permease | -1.56 |
| AFUA_7G01200 |  | Putative aspergillopepsin | -1.54 |
| AFUA_6G03350 |  | Putative GNAT family N-acetyltransferase | -1.52 |
| AFUA_6G03330 |  | Hypothetical protein | -1.50 |
| AFUA_2G05280 |  | F-box domain protein | -1.49 |
| AFUA_3G01850 |  | Porphyromonas-type peptidyl-arginine deiminase superfamily | -1.48 |
| AFUA_5G02860 |  | Integral membrane protein | -1.48 |
| AFUA_1G16020 |  | Hypothetical protein | -1.47 |
| AFUA_7G01410 |  | UPF0183 domain protein | -1.46 |
| AFUA_8G07225 |  | Hypothetical protein | -1.45 |
| AFUA_5G10240 |  | Hypothetical protein | -1.44 |
| AFUA_3G11900 |  | Oxidoreductase, zinc-binding | -1.44 |
| AFUA_6G11020 |  | 3-hydroxyisobutyrate dehydrogenase | -1.42 |
| AFUA_4G06110 |  | Hypothetical protein | -1.42 |
| AFUA_2G13230 |  | Universal stress protein family domain protein | -1.42 |
| AFUA_8G05690 |  | Acetyltransferase, GNAT family family | -1.41 |
| AFUA_8G01000 |  | Ankyrin repeat protein | -1.40 |
| AFUA_2G17460 | *AldH12* | Putative aldehyde dehydrogenase | -1.39 |
| AFUA_4G12000 |  | Putative phosphatidylinositol phospholipase C | -1.39 |
| AFUA_3G03700 |  | Putative MFS sugar transporter | -1.38 |
| AFUA_4G09850 |  | Hypothetical protein | -1.38 |
| AFUA_3G01650 |  | Putative WSC domain protein | -1.38 |
| AFUA_8G00962 |  | Putative cytochrome P450 | -1.38 |
| AFUA_3G00840 |  | Putative FAD-dependent oxygenase | -1.37 |
| AFUA_1G15180 |  | Hypothetical protein | -1.36 |
| AFUA_4G00830 |  | Putative MFS peptide transporter | -1.36 |
| AFUA_2G00220 |  | Putative aminopeptidase | -1.35 |
| AFUA_2G12310 |  | Putative HLH transcription factor | -1.35 |
| AFUA_2G16930 |  | Putative succinate:fumarate antiporter | -1.35 |
| AFUA_4G01360 |  | MFS transporter of unkown specificity | -1.31 |
| AFUA_3G12710 |  | Hypothetical protein | -1.31 |
| AFUA_2G17450 |  | Putative 3-hydroxyanthranilate 3,4-dioxygenase | -1.30 |
| AFUA_2G11350 | *Kat1* | Putative peroxisomal 3-ketoacyl-coA thiolase | -1.30 |
| AFUA_8G02170 |  | Putative C6 finger domain protein | -1.29 |
| AFUA_1G14800 |  | Hypothetical protein | -1.29 |
| AFUA_6G12230 |  | Hypothetical protein | -1.28 |
| AFUA_3G11480 | *Auh* | Putative mitochondrial methylglutaconyl-CoA hydratase | -1.28 |
| AFUA_6G08760 | *PrnD* | Proline oxidase | -1.28 |
| AFUA_1G12910 |  | Putative ABC fatty acid transporter | -1.28 |
| AFUA_6G14350 |  | Putative C6 transcription factor | -1.27 |
| AFUA_2G17440 |  | Putative 2-amino-3-carboxymuconate-6-semialdehyde decarboxylase | -1.27 |
| AFUA_8G00610 | *mas1* | Putative cell surface protein | -1.27 |
| AFUA_4G14300 |  | Putative dynamin family GTPase | -1.26 |
| AFUA_5G11840 |  | Putative protein kinase | -1.26 |
| AFUA_2G17470 |  | Putative L-PSP endoribonuclease family protein | -1.26 |
| AFUA_4G11710 |  | Oxidoreductase, zinc-binding dehydrogenase family superfamily | -1.26 |
| AFUA_5G13310 |  | Putative C6 transcription factor | -1.25 |
| AFUA_2G00320 | *erg3* | Putative sterol delta 5,6-desaturase | -1.24 |
| AFUA_8G02180 |  | Putative GABA permease | -1.24 |
| AFUA_5G14730 | *AKR13* | Putative aldo-keto reductase | -1.24 |
| AFUA_2G00330 |  | Putative beta-alanine synthase | -1.23 |
| AFUA_2G08090 |  | Hypothetical protein | -1.23 |
| AFUA_7G01680 |  | Hypothetical protein | -1.23 |
| AFUA_5G11890 |  | Rho guanyl nucleotide exchange factor | -1.23 |
| AFUA_5G01662 |  | Pathway-specific regulatory protein | -1.23 |
| AFUA_3G12780 |  | Putative C6 transcription factor | -1.23 |
| AFUA_1G16010 |  | Hypothetical protein | -1.22 |
| AFUA_6G08860 |  | Sugar isomerase, KpsF/GutQ | -1.21 |
| AFUA_7G03770 |  | Hypothetical protein | -1.20 |
| AFUA_4G07040 | *ctsD* | Putative secreted aspartic-type endopeptidase | -1.19 |
| AFUA_1G13750 | *Rpn4* | Putative C2H2 transcription factor | -1.19 |
| AFUA_4G14440 |  | Putative NRPS-like enzyme | -1.19 |
| AFUA_2G07720 |  | Putative cytochrome b5 | -1.19 |
| AFUA_3G02270 | *cat1* | Mycelial catalase | -1.19 |
| AFUA_2G01300 |  | Hypothetical protein | -1.19 |
| AFUA_4G08580 | *prx1* | Mitochondrial peroxiredoxin | -1.18 |
| AFUA_3G01330 |  | Class II aldolase/adducin domain protein | -1.18 |
| AFUA_5G00790 |  | Putative ABC multidrug transporter | -1.18 |
| AFUA_4G14460 | *tpcM* | Methyltransferase | -1.18 |
| AFUA_6G08740 |  | Putative 3-dehydroshikimate dehydratase | -1.17 |
| AFUA_1G14090 |  | Histidinol-phosphate aminotransferase | -1.17 |
| AFUA_2G00240 |  | Hypothetical protein | -1.17 |
| AFUA_6G02390 |  | Thioesterase family protein | -1.17 |
| AFUA_1G14610 | *Smp2* | Putative lipin | -1.16 |
| AFUA_5G06300 |  | Putative peroxisomal membrane protein | -1.16 |
| AFUA_6G00690 |  | Hypothetical protein | -1.16 |
| AFUA_8G04540 |  | Putative C6 transcription factor | -1.16 |
| AFUA_5G08090 | *pyroA* | Putative pyridoxine biosynthesis protein | -1.16 |
| AFUA_3G12770 |  | Putative nucleoside-diphosphate-sugar epimerase | -1.15 |
| AFUA_1G15910 |  | Putative C6 transcription factor RosA-like | -1.15 |
| AFUA_4G06190 |  | Putative fungal specific transcription factor | -1.15 |
| AFUA_5G07860 |  | Putative phosphatase family protein | -1.15 |
| AFUA_3G05730 |  | Nicotinate mononucleotide pyrophosphorylase | -1.15 |
| AFUA_6G08155 |  | Putative pyroglutamyl peptidase type I | -1.15 |
| AFUA_2G02070 |  | CCR4-NOT transcription complex, subunit 3 | -1.15 |
| AFUA_3G10940 | *Pex19* | Putative peroxisomal membrane protein receptor | -1.14 |
| AFUA_7G06120 |  | Putative transmembrane transporter | -1.14 |
| AFUA_2G05240 |  | Hypothetical protein | -1.14 |
| AFUA_2G03500 |  | Putative sugar transporter | -1.14 |
| AFUA_7G06350 | *phoE* | Putative phosphate transporter, phoB-regulated | -1.14 |
| AFUA_1G05860 |  | Hypothetical protein | -1.14 |
| AFUA_2G01640 |  | Tryptophanyl-tRNA synthetase | -1.14 |
| AFUA_1G16650 |  | Hypothetical protein | -1.13 |
| AFUA_5G03350 |  | Putative glutamine dependent NAD+ synthetase | -1.13 |
| AFUA_8G02570 |  | Putative Baeyer-Villiger monooxygenase (BVMO), flavin-binding monooxygenase | -1.13 |
| AFUA_1G15940 |  | Auxin Efflux Carrier superfamily | -1.13 |
| AFUA_5G08020 | *Penr2* | Putative helix-loop-helix DNA binding protein | -1.13 |
| AFUA_3G01920 |  | Putative acyl-CoA dehydrogenase | -1.13 |
| AFUA_6G03340 |  | Hypothetical protein | -1.12 |
| AFUA_2G12450 |  | Putative hydroxymethylglutaryl-CoA lyase | -1.12 |
| AFUA_2G11110 | *Smc2* | Putative nuclear condensin complex subunit | -1.12 |
| AFUA_4G09560 | *zrfC* | Zinc-regulated transporter | -1.11 |
| AFUA_1G02840 |  | Hypothetical protein | -1.11 |
| AFUA_2G01330 | *Rrp46* | Putative exosome complex subunit | -1.11 |
| AFUA_4G06380 |  | Putative sterol carrier protein | -1.10 |
| AFUA_4G09050 | *atg1* | Putative serine/threonine-protein kinas | -1.10 |
| AFUA_3G10780 |  | Hypothetical protein | -1.10 |
| AFUA_3G13100 |  | Hypothetical protein | -1.10 |
| AFUA_5G04310 | *pmp47* | Putative peroxisome membrane protein | -1.09 |
| AFUA_3G08430 |  | Putative mitochondrial phosphate carrier protein | -1.09 |
| AFUA_4G06510 |  | Hypothetical protein | -1.09 |
| AFUA_3G10100 | *Srp2* | Putative pre-RNA splicing factor | -1.09 |
| AFUA_1G02620 |  | Hypothetical protein | -1.09 |
| AFUA_8G00760 |  | Putative cytochrome P450 | -1.08 |
| AFUA_3G06740 | *Gal4* | Putative C6 transcription factor | -1.08 |
| AFUA_2G11340 |  | Putative ML domain protein | -1.08 |
| AFUA_5G02850 |  | Hypothetical protein | -1.08 |
| AFUA_1G00530 |  | Thermoresistant gluconokinase family protein | -1.08 |
| AFUA_3G00340 |  | Putative glycosyl hydrolase | -1.07 |
| AFUA_2G04100 |  | Hypothetical protein | -1.06 |
| AFUA_3G00820 |  | Hypothetical protein | -1.06 |
| AFUA_6G03740 | *DmtA* | C-5 cytosine methyltransferase | -1.06 |
| AFUA_1G03760 |  | DUF726 domain protein | -1.06 |
| AFUA_6G03550 |  | Hypothetical protein | -1.06 |
| AFUA_2G12970 |  | Putative peroxisomal membrane anchor protein | -1.06 |
| AFUA_6G00412 |  | Putative amino acid permease | -1.06 |
| AFUA_6G03670 |  | Putative biotin synthase | -1.06 |
| AFUA_2G14280 | *KlpA* | Putative kinesin family protein | -1.06 |
| AFUA_2G14490 |  | Putative endoglucanase | -1.06 |
| AFUA_2G16090 |  | Karyopherin alpha subunit | -1.06 |
| AFUA_8G02670 |  | Hypothetical protein | -1.05 |
| AFUA_7G02010 |  | Indoleamine 2,3-dioxygenase family protein | -1.05 |
| AFUA_1G14620 |  | Fibronectin type III domain protein | -1.05 |
| AFUA_2G01350 | *Utr1* | Putative NAD+ kinase | -1.04 |
| AFUA_7G05960 |  | Putative C2H2 finger domain protein | -1.04 |
| AFUA_6G13346 |  | Hypothetical protein | -1.04 |
| AFUA_1G02530 |  | Putative MFS sugar transporter | -1.03 |
| AFUA_2G08950 |  | Putative isochorismatase family hydrolase | -1.03 |
| AFUA_8G02150 |  | Putative acetylornithine aminotransferase | -1.03 |
| AFUA_3G12970 | *pns1* | Putative choline transporter-like | -1.03 |
| AFUA_2G04570 |  | BNR/Asp-box repeat domain protein | -1.03 |
| AFUA_3G10000 | *pkaR* | cAMP-dependent protein kinase regulatory subunit | -1.03 |
| AFUA_2G00540 |  | Putative carboxyphosphonoenolpyruvate phosphonomutase | -1.03 |
| AFUA_4G04415 |  | IBR domain protein | -1.02 |
| AFUA_2G11840 | *cyc8* | Transcriptional corepressor | -1.02 |
| AFUA_8G04480 |  | Hexose transporter protein | -1.02 |
| AFUA_7G04610 |  | Ulp1 protease family protein | -1.02 |
| AFUA_6G09650 | *gliJ* | Membrane dipeptidase | -1.02 |
| AFUA_6G13140 |  | 3,4-dihydroxy-2-butanone 4-phosphate synthase | -1.01 |
| AFUA_4G07180 |  | Hypothetical protein | -1.01 |
| AFUA_1G15630 |  | Hypothetical protein | -1.01 |
| AFUA_2G16985 |  | Integral membrane protein | -1.01 |
| AFUA_4G00960 | *pfkA* | Putative 6-phosphofructokinase alpha subunit | -1.01 |
| AFUA_3G02530 |  | Protein similar to polyketide synthases (PKS-like), encoded in a predicted secondary metabolite gene cluster | -1.01 |
| AFUA_2G09030 | *dppV* | Secreted dipeptidyl-peptidase | -1.01 |
| AFUA_1G14400 |  | Putative hydroxyisocaproate dehydrogenase | -1.01 |
| AFUA_6G03510 |  | Putative flavin containing polyamine oxidase | -1.01 |
| AFUA_1G09300 |  | Hypothetical protein | -1.01 |
| AFUA_5G01248 |  | Hypothetical protein | -1.00 |

**Table S6. Protein-protein interactions (PPI) in degree sorted network of 328 down-regulated DEGs and 67 up-regulated DEGs into 22 clusters. KEGG pathway functional enrichment was described for each cluster.**

| Clusters | Number of nodes | Number of edges | KEGG pathway  functional enrichment description | PPI enrichment  p-value |
| --- | --- | --- | --- | --- |
| **Total** | **395** | **1113** |  | **< 1.0e-16** |
| Cluster 1 | 29 | 258 | Peroxisome  Biosynthesis of unsaturated fatty acids  beta-Alanine metabolism  Fatty acid degradation  Propanoate metabolism | < 1.0e-16 |
| Cluster 2 | 23 | 124 | Cell cycle  Meiosis | < 1.0e-16 |
| Cluster 3 | 8 | 23 | Glyoxylate bypass  Carbon metabolism  Metabolic pathways | < 1.0e-16 |
| Cluster 4 | 7 | 11 | Alanine, aspartate and glutamate metabolism | 3e-15 |
| Cluster 5 | 6 | 11 | Valine, leucine and isoleucine degradation  Fatty acid degradation | < 1.0e-16 |
| Cluster 6 | 6 | 11 | Tyrosine metabolism | < 1.0e-16 |
| Cluster 7 | 5 | 4 | NA | 8.33e-11 |
| Cluster 8 | 5 | 6 | NA | 5.51e-11 |
| Cluster 9 | 4 | 3 | Taurine and hypotaurine metabolism | 2.23e-06 |
| Cluster 10 | 4 | 4 | Fatty acid degradation, metabolism, biosynthesis  Peroxisome | 2.07e-08 |
| Cluster 11 | 4 | 5 | Propanoate metabolism  Valine, leucine and isoleucine degradation | 7.44e-08 |
| Cluster 12 | 3 | 2 | Histidine metabolism  Ascorbate and aldarate metabolism  beta-Alanine metabolism  Pantothenate and CoA biosynthesis  Tryptophan metabolism | 0.00188 |
| Cluster 13 | 3 | 3 | alpha-Linolenic acid metabolism  Biosynthesis of unsaturated fatty acids  Valine, leucine and isoleucine degradation  Fatty acid degradation, metabolism  Peroxisome | 7.09e-06 |
| Cluster 14 | 3 | 2 | Pyruvate metabolism | 0.000212 |
| Cluster 15 | 3 | 2 | Butanoate metabolism  Alanine, aspartate and glutamate metabolism  Valine, leucine and isoleucine degradation | 0.000117 |
| Cluster 16 | 3 | 2 | Glycolysis / Gluconeogenesis  Biosynthesis of secondary metabolites | 4.62e-05 |
| Cluster 17 | 3 | 2 | Meiosis | 2.05e-06 |
| Cluster 18 | 3 | 2 | NA | 0.000107 |
| Cluster 19 | 3 | 2 | Ether lipid metabolism  Glycerophospholipid metabolism | 0.002350 |
| Cluster 20 | 3 | 3 | Meiosis | 4.57e-13 |
| Cluster 21 | 3 | 2 | NA | 2.45e-05 |
| Cluster 22 | 3 | 2 | Steroid biosynthesis | 0.000109 |

NA: Not Available

**Table S7. Composition of media (g/L)**

| Composition | NP (TSB) | YPD | MRS | LB | M17 | MH | BHI | CZ | PDB | MEB | MM |
| --- | --- | --- | --- | --- | --- | --- | --- | --- | --- | --- | --- |
| Glucose | 2.5 | 20 | 20 |  |  |  | 2 |  | 20 | 6 | 10 |
| Maltose |  |  |  |  |  |  |  |  |  | 1.8 |  |
| Sucrose |  |  |  |  |  |  |  | 30 |  |  |  |
| Yeast extract |  | 10 | 5 | 5 | 2.5 |  |  |  |  | 1.2 |  |
| Beef extract |  |  | 10 |  | 5 | 2 |  |  |  |  |  |
| Malt extract |  |  |  |  |  |  |  |  |  | 6 |  |
| Peptone |  | 20 | 10 |  |  |  |  |  |  |  |  |
| Soy peptone |  |  |  |  | 5 |  |  |  |  |  |  |
| Tryptone |  |  |  | 10 |  |  |  |  |  |  |  |
| Pancreatic digest of casein | 17 |  |  |  | 5 |  |  |  |  |  |  |
| Acid hydrolysate of casein |  |  |  |  |  | 17.5 |  |  |  |  |  |
| Pancreatic digest of gelatin |  |  |  |  |  |  | 10 |  |  |  |  |
| Peptic digest of soybean | 3 |  |  |  |  |  |  |  |  |  |  |
| Starch |  |  |  |  |  | 1.5 |  |  |  |  |  |
| Potato Starch |  |  |  |  |  |  |  |  | 4 |  |  |
| Brain heart infusion |  |  |  |  |  |  | 17.5 |  |  |  |  |
| Monopotassium phosphate |  |  |  |  |  |  |  |  |  |  | 1.52 |
| Dipotassium phosphate | 2.5 |  |  |  |  |  |  | 1 |  |  |  |
| Potassium chloride |  |  |  |  |  |  |  | 0.5 |  |  | 0.52 |
| Disodium phosphate |  |  | 2 |  |  |  | 2.5 |  |  |  |  |
| Disodium-β-glycerophosphate |  |  |  |  | 19 |  |  |  |  |  |  |
| Magnesium sulfate |  |  | 0.1 |  | 0.25 |  |  | 0.5 |  |  | 0.52 |
| Ferrous sulfate |  |  |  |  |  |  |  | 0.01 |  |  | 0.005 |
| Copper sulfate |  |  |  |  |  |  |  | 0.005 |  |  | 0.0016 |
| Zinc sulfate |  |  |  |  |  |  |  | 0.001 |  |  | 0.022 |
| Ammonium molybdate |  |  |  |  |  |  |  |  |  |  | 0.0011 |
| Manganese dichloride |  |  |  |  |  |  |  |  |  |  | 0.005 |
| Cobalt chloride |  |  |  |  |  |  |  |  |  |  | 0.0016 |
| Sodium chloride | 5 |  |  | 10 |  |  | 5 |  |  |  |  |
| Sodium acetate |  |  | 5 |  |  |  |  |  |  |  |  |
| Sodium nitrate |  |  |  |  |  |  |  | 3 |  |  | 6 |
| EDTA disodium salt |  |  |  |  |  |  |  |  |  |  | 0.05 |
| Polysorbate 80 |  |  | 1 |  |  |  |  |  |  |  |  |
| Ascorbic acid |  |  |  |  | 0.5 |  |  |  |  |  |  |
| Boric acid |  |  |  |  |  |  |  |  |  |  | 0.011 |

**Table S8. Composition of NP producing medium**

| **Component (NP producing medium)** | **Concentration (g/L)** |
| --- | --- |
| Pancreatic digest of casein | 17 |
| Papaic digest of soybean | 3 |
| Dextrose | 2.5 |
| Sodium chloride | 5 |
| Dipotassium phosphate | 2.5 |

| **Organic solvents** | | **Polarity** | **DI** |
| --- | --- | --- | --- |
| Ethyl acetate | C_4_H_8_O_2_ | 4.4 | 6.02 |
| Chloroform | CHCl_3_ | 4.1 | 4.81 |
| Tetrahydrofuran | C_4_H_8_O | 4.0 | 7.58 |
| Dichloromethane | CH_2_Cl_2_ | 3.1 | 9.1 |
| Diethyl ether | (C2H5)2O | 2.8 | 4.33 |
| Toluene | C_6_H_5_CH_3_ | 2.4 | 2.38 |
| EMW | Ethyl acetate: Met2.4hanol: water  = 10: 1.35: 1 (Polar, Neutral) | | |
| CEF | Chloroform: Ethyl acetate: Formic acid  = 5: 4: 1 (Intermediate polarity, Acidic) | | |
| BEA | Benzene: Ethanol: Ammonia hydroxide  = 9: 1: 0.1 (Nonpolar, Basic) | | |

**Table S9. Organic solvents for extraction of NP based on polarity**
